# Screening of short-day onions for resistance to *Stemphylium* leaf blight in the seed-to-bulb stage (stage I) and bulb-to-seed stage (stage II)

**DOI:** 10.3389/fpls.2022.1063685

**Published:** 2022-11-16

**Authors:** Rahul Chandel, Deeba Kamil, Shrawan Singh, Amrender Kumar, Rumit Patel, Priyanka Verma, Masochon Zimik, Anil Khar

**Affiliations:** ^1^ Division of Vegetable Science, The Indian Council of Agricultural Research (ICAR)-Indian Agricultural Research Institute, Delhi, India; ^2^ Division of Plant Pathology, The Indian Council of Agricultural Research-Indian Agricultural Research Institute, Delhi, India; ^3^ Agricultural Knowledge Management Unit, The Indian Council of Agricultural Research-Indian Agricultural Research Institute, Delhi, India; ^4^ Department of Agricultural Biotechnology, Anand Agricultural University, Anand, Gujarat, India

**Keywords:** *Allium cepa* L., resistance, *Stemphylium* leaf blight, heritability, enzymes

## Abstract

*Stemphylium* leaf blight, caused by *Stemphylium vesicarium*, is a very important fungal disease in onions since its epidemics are able to affect both the bulb yield and the seed quality. The aim of this study was to screen onion genotypes at stage I (seed to bulb) and further screen the identified resistant and susceptible genotypes at stage II (bulb to seed). One hundred and fifty-seven genotypes were screened against SLB under artificially inoculated field conditions. Results revealed a significant variation among the morphological and biochemical traits studied. Correlation studies revealed a significant and negative correlation between percent disease incidence (PDI), pseudostem width, neck thickness, and dry matter. Fifteen genotypes were identified as moderately resistant, and the rest were categorized as susceptible. Bulbs of the genotypes, identified as moderately resistant, were again screened for resistance in stage II. All the genotypes were categorized as moderately susceptible. Biochemical analysis revealed that total foliar phenol content, pyruvic acid, catalase, and peroxidase increased up to 20 days after inoculation (DAI) and thereafter declined. Protein content was highest in the initial stage and declined at 10, 20, and 30 DAI. The higher biochemical activity was observed in moderately resistant category genotypes compared with the susceptible ones. Correlation analysis showed a highly significant and negative correlation of PDI with total foliar phenol content (TFPC), pyruvic acid, catalase, peroxidase, and protein content. To conclude, it was observed that screening against SLB should be done at both the stages (stage I and Stage II) to identify resistant onion genotypes. Direction selection for genotypes with high dry matter, higher phenols, and enzymes may be an alternative pathway to select genotypes for a robust resistance breeding program.

## Introduction

Onion (*Allium cepa* L.), an indispensable vegetable, is used as the main ingredient in almost every cuisine throughout the world. It belongs to the family *Amaryllidaceae* and is a high-value bulbous cum spice vegetable crop. Onions contain diverse bioactive compounds, such as organosulfurs, flavonols, polysaccharides, phenolic compounds, and saponins which exhibit bactericidal, anticancer, and antioxidant activities (Javadzadeh et al., 2009; Zhao et al., 2021). India is the largest producer of onion in the world with an output of 23.73 million tons from an area of 1.43 million ha and accounts for more than 30% of total world production (FAOSTAT, 2020). In 2020, India exported 1.57 mMT of fresh onions to the world with a forex earning value of US$ 378.49 million (APEDA, 2020). Despite being the largest producer, India is far behind in terms of productivity (18.64 t/ha) when compared with other onion-producing countries like the Republic of Korea (79.61 t/ha), USA (71.09 t/ha), and Australia (54.66 t/ha) (FAOSTAT, 2020). Various biotic (purple blotch, *Stemphylium* blight, *Anthracnose*, *Fusarium* basal rot, thrips, iris yellow spot virus, etc.) and abiotic (high rainfall, drought, salinity, temperature stress) factors are known to severely hamper the productivity and nutritional potential of the onion, the most prominent being the diseases caused by phytopathogens. Onions are highly susceptible to several foliar, bulb, and root pathogens that tremendously reduce the yield and nutritive value of the crops (Khar et al., 2022).

*Stemphylium vesicarium* (Wallr.) E. Simmons is the most common destructive foliar fungus which causes *Stemphylium* leaf blight disease (SLB) of onions worldwide. This pathogen also infects other economically important crops of the *Amaryllidaceae* family such as garlic (*Allium sativum* L.) and leek (*Allium ampeloprasum* L.). In particular, *Stemphylium vesicarium* is a hemibiotrophic fungal pathogen with a broad range of host crop species such as vegetable, field, ornamental, and tree fruit crops (Hay et al., 2021). SLB has emerged as a major bottleneck for achieving the full genetic potential of both bulb and seed crops (Dangi et al., 2019). In terms of yield and quality, SLB is reported to cause 90% losses in onion (Raghavendra Rao and Pavgi 1975; Miller et al., 1978; Lorbeer, 1993). It causes severe necrosis and defoliation of the foliage resulting in the reduction of the photosynthetic area. Consequently, bulbs remain small sized and underdeveloped, and upon maturity, tops of bulbs may not lodge which later undergo decay by soil microbes (Raghavendra Rao and Pavgi, 1975). In severe conditions, SLB can also result in premature mortality of plants (Hoepting, 2017). Infection on onion leaves can occur at wide ranges of temperature from 10°C to 26°C (Basallote-Ureba et al., 1999). A high relative humidity (RH) of more than 85%, temperatures ranging from 18°C to 22°C, and leaf wetness for at least 8 h are favorable for disease development in onion crops (Suheri and Price, 2000). Disease severity is more on seed crops compared with bulb crops (Tomaz and Lima, 1988), and sometimes it can cause up to 100% loss in seed production (Singh et al., 1992).

SLB is primarily managed through the frequent application of fungicides (single-site mode of action) that can lead to resistance development among pathogen populations (Hay et al., 2019). Insensitivity or lesser efficacy of common and popular fungicides for SLB management has been reported (Hay et al., 2019). A higher level of pesticide residues in onions is also a major concern for onion exports. India has a huge potential for exporting onion to markets of European nations and countries like USA and Canada. Indian onion must possess certain standards especially in terms of quality and pesticide residues that should not be more than the prescribed levels to capture these markets. Onion growers are in need of resistant cultivars as an important preventative step of integrated plant management (IPM) so that the best quality and minimum pesticide residue can be ensured in export-oriented onion cultivation. Resistance/tolerance and susceptibility among plants are mediated by a complex network of biochemical and molecular events. Plants intrinsically contain natural chemical compounds such as enzymes, phenols, sugars, and other secondary metabolites like alkaloids, cucurbitacin, flavonoids, glycosides, and terpenoids that prevent the proliferation of disease-causing pathogens inside them (Koul and Walia, 2009; Hussein et al., 2018).

Currently, not a single commercial onion variety resistant to SLB disease is available in the market worldwide. In previous studies, it has been found that with different degrees of susceptibility, almost all the commercial varieties succumb to *S. vesicarium* infection (Foster et al., 2019; Dangi et al., 2019; Hay et al., 2021). Several workers have reported a moderate level of resistance in their studies against *Stemphylium* leaf blight. Sharma and Sain (2003) developed a variety RO-1 which was found to be moderately resistant against blight. In another study, three moderately resistant cultivars were reported by Behera et al. (2013). Dangi et al. (2019) reported two cultivars, Red Creole 2 (onion) and Pusa Soumya (Bunching-type onion), as moderately resistant. Genetic studies revealed a possible dominant gene control of the resistance trait, but F_2_ generation did not follow the Mendelian segregation because of sterility issues in F_1_ hybrids (Pathak et al., 2001).

SLB creates havoc as a foliar disease in both the seed-to-bulb stage (stage I) and bulb-to-seed stage (stage II). Hence, the aim of this study was i) to screen the genotypes, in stage I and stage II, under artificially inoculated conditions for *Stemphylium* leaf blight, ii) to assess the morphological and biochemical parameters, iii) to identify whether genotypes resistant to SLB in stage I remain resistant in stage II also, and iv) to assess the relationship between PDI and biochemical traits.

## Materials and methods

### Isolation, identification, and purification of pathogens

Onion leaves showing SLB symptoms were collected from the field of the Division of Vegetable Science, ICAR-Indian Agricultural Research Institute, New Delhi. The infected leaves were washed properly under running tap water to remove dirt and soil. Surface sterilization of the diseased leaves was performed by immersing them in sodium hypochlorite (NaOCl) solution at 2% (v/v) solution for 1–2 min and then washed twice with sterile distilled water. After drying on sterilized tissue paper, leaves were fragmented into small pieces of approx. 5–6 mm^2^. Fragments of leaves were cultured on sterile potato dextrose agar media (PDA, HiMedia) in plastic petri plates (90 mm dia × 15 mm depth) and incubated at 25 ± 2°C for 24–48 h. Emerging fungal colonies were closely monitored for their growth pattern, and with the help of a sterile needle, the hyphal tip of the fungal colony was subcultured on the fresh potato dextrose agar media. These subcultured Petri plates were kept at 25 ± 2°C temperature for 7–10 days under a 14-h light and 10-h dark period. The fungus was identified based on morphological characteristics (conidia and conidiophore shape and size; Ellis, 1971) and the internal transcribed spacer (ITS) sequence. DNA was extracted from the fungal mycelial mat using the CTAB method (Cullings, 1992), and the ITS region was amplified using primers ITS1 (5′-TCCGTAGGTGAACCTGCGG-3′) and ITS4 (5′-TCCTCCGCTTATTGATATGC-3′) (White et al., 1990). The amplified product was sequenced directly by outsourcing (Barcode Biosciences, New Delhi) and identified using NCBI BLAST. The sequence was submitted to NCBI, and the GenBank number was obtained. The pure culture of *S. vesicarium* was utilized for the preparation of 1 × 10^5^ ml^−1^ spore suspensions for inoculation.

### Field screening (seed-to-bulb stage) through artificial inoculation and experimental layout

The field screening experiment through artificial inoculation was conducted by spraying the spore suspension in the experimental field of the Division of Vegetable Science, ICAR-IARI, New Delhi (77°09′27″E, 28°38′23″N, and altitude 228.61 m above the mean sea level). Seeds of 157 onion accessions, consisting of breeding lines, landraces, open-pollinated varieties (OPVs), and hybrids (**Supplementary Table 1**), were sown in the nursery during the second week of October 2020. The transplanting of plants to the experimental plot was done on the third week of December 2020 in a randomized complete block design with three replications. Sixty plants per accession were transplanted with a row spacing of 15 cm and a plant spacing of 10 cm. Recommended cultural practices were followed, and a nitrogen: phosphorus: potassium (NPK) fertilizer was applied at 110:40:60 kg/ha. Plants of highly susceptible accession number AKON068 were planted in every third row and around the border of the experiment plot for an additional source of inoculum for infection. For ensuring uniform soil moisture throughout the experimental plot, flood irrigation was applied at 7–10-day intervals throughout the experimentation period. Manual removal of weeds was carried out at 15-day intervals until the neck-fall stage. The experimental plot was surrounded by a 2.50-m-high translucent plastic sheet, and the application of fungicides was restricted in and around the experimentation site.

### Morphological and biochemical traits under stage I

Morphological traits such as plant height (PH, cm), leaf length (LL, cm), leaf width (LW, cm), number of leaves per pseudostem (NOL), pseudostem length (PsL, cm), pseudostem width (PsW, mm), polar diameter (P, mm), equatorial diameter (E, mm), neck thickness (N, mm), bolting percentage, days to maturity (DTM), average bulb weight (ABW, g), gross yield (GW, t/ha), and marketable yield (MY, t/ha) were recorded. Gross yield was calculated according to the following formula: Yield (tons/ha) = (Yield in kg per plot × 666.67)/Number of plants planted. Marketable yield was calculated using the same formula, and bulbs having an equatorial diameter greater than 3.50 cm were counted as marketable. The dry matter (DM) of the bulbs was assessed according to Nieuwhof et al. (1973) and expressed in percentage (%). Total soluble solids (T.S.S) were determined by using a hand refractometer (model, PAL-3) and expressed in °B.

### Screening the bulb-to-seed stage under controlled conditions (stage II)

Genotypes showing a moderately resistant and susceptible reaction were identified under field conditions (**Supplementary Table 2**). Bulbs of the same genotypes were screened for *Stemphylium* blight under controlled conditions since SLB is also a devastating disease for seed crop too. The experiment was conducted inside the automated greenhouse of the Division of Vegetable Science, ICAR-Indian Agricultural Research Institute, New Delhi. This experiment comprised 20 previously identified genotypes (15 moderately resistant, four susceptible, one highly susceptible) during the field screening. The experiment was laid out in a completely randomized design with two replications. Five bulbs of each accession were planted in each pot filled with sterilized cocopeat, perlite, and vermiculite in the ratio of 2:1:1, respectively. The onion plants were kept at 18°C–22°C temperature under ambient light conditions and watered every 3–5 days. Plants were fertilized after 15 days of planting. Thereafter, every week a water-soluble fertilizer (NPK 19:19:19) at the rate of 5 g/l was applied.

### Inoculation of fungus *S. vesicarium* under field and controlled conditions

For field screening, onion plants were inoculated 60 days after transplanting. A conidial suspension of 1 × 10^5^ ml^−1^ was prepared by serial dilution technique and sprayed using a knapsack sprayer in the evening followed by flood irrigation to maintain the optimum relative humidity (RH). Onion plant leaves were sprayed with fine mist of water twice after 8–10 h of inoculum spraying to maintain leaf wetness for facilitating better conidium germination. The field was irrigated repeatedly at 2-day intervals until the disease appeared. The fungus was re-isolated from the infected leaves, and its identity was confirmed through morphological observations. In greenhouse screening, a similar concentration of conidial suspension was used as mentioned. For maintenance, a relative humidity of<90% and leaf wetness for 8–10 h using automated misters were applied.

### Disease assessment (scoring the incidence of disease)

Scoring of the disease incidence was done by using a 0–5-point scale (**Table 1**) (Sharma, 1986). Disease assessment was done by taking 10 plants per accession and per replicate into consideration at an interval of 10 days after the inoculation and repeated thrice. The infected areas of each leaf were observed visually. Percent disease incidence (PDI) was calculated (Wheeler, 1969) using the following formula:

**Table 1:** *Stemphylium* leaf blight interactions and phenotypic classes used for the evaluation of onion genotypes (Pathak et al., 1986).

Disease severity percentage (%)	Disease reaction	Characteristics	Disease score
<5.0	Immune	No symptom	0
5.1-10.0	Resistant	Few spots toward the tip, covering less than 10% leaf area	1
10.1-20.0	Moderately resistant	Several dark brown patches covering less than 20% leaf area	2
20.1-40.0	Moderately susceptible	Large patches with paler outer zone, covering up to 40% leaf area	3
40.1-60.0	Susceptible	Long streaks covering up to 75% leaf area or breaking of leaves from the center	4
>60.1	Highly susceptible	Complete drying of the leaves or breaking of the leaves from the base	5


$$PDI=\frac{N_{1}\times 1+N_{2}\times 2+N_{3}\times 3+N_{4}\times 4+N_{5}\times 5}{Total\;number\;of\;observed\;leaves\;\times Maximum\;grade}\;\times 100$$

where N_1_ to N_5_ represent the total number of leaves falling under the 1–5 scales, respectively. Based on their PDI values, each onion accession was classified into six disease reaction classes: immune (I<5), resistant (5.1 ≤R≥ 10.0), moderately resistant (10.1 ≤MR≥ 20.0), moderately susceptible (20.1 ≤MS≥ 40.0), susceptible (40.1 ≤S≥ 60.0), and highly susceptible (HS >60.0) (Pathak et al., 1986). The area under the disease progress curve (AUDPC) was calculated using the trapezoidal method according to a formula given by Madden et al. (2007).


$$AUDPC=\overset{n-1}{\underset{i=1}{\sum }}\frac{\left(y_{i}+y_{i+1}\right)}{2}\left(t_{i+1}-t_{i}\right)$$

where *yi* is an assessment of a disease (percentage) at the *i*th observation, *ti* is the time (in days) at the *i*th observation, and *n* is the total number of observations.

### Biochemical characterization under stage II

For biochemical analyses, leaves were collected from onion bulbs grown in pots under greenhouse conditions. To perform biochemical analyses in leaf tissues, leaf samples were frozen in liquid nitrogen. Leaf samples were collected before inoculation and later on 10-day intervals at 10, 20, and 30 days after inoculation with the *S. vesicarium* pathogen.

### Total foliar phenol content

The total phenolic content of onion leaf was estimated spectrophotometrically using the Folin–Ciocalteu (FC) reagent method developed by Singleton and Rossi (1965). Absorbance was taken using a UV-1900i UV-Vis double-beam spectrophotometer (SHIMADZU, Japan) at 750 nm. Total phenol content was expressed in ‘mg gallic acid equivalent (GAE)’ per g of fresh weight.

### Pyruvic acid

Pyruvic acid was estimated spectrophotometrically through the method of Anthon and Barrett (2003). Standards were prepared by using sodium pyruvate solution. The reading was carried out in a spectrophotometer at 515 nm using the UV-1900i UV-Vis double-beam spectrophotometer (SHIMADZU, Japan). Pyruvic acid was expressed as µmole g^−1^ sample.

### Catalase

Catalase activity estimation was done according to Aebi (1984). The H_2_O_2_ oxidation was assayed spectrophotometrically at 240 nm every 30 s for 2 min, and activity was computed by calculating the amount of H_2_O_2_ decomposed. A standard curve drawn with the known concentration of H_2_O_2_ was used to determine the initial and final contents of hydrogen peroxide. Catalase activity was measured in μmol H_2_O_2_/min/mg protein.

### Guaiacol peroxidase

The peroxidase activity was assayed by using guaiacol as the substrate by following the method described by Chance and Maehly (1955). The guaiacol peroxidase activity was determined spectrophotometrically by measuring the increase in absorbance at 470 nm by the conversion of guaiacol to tetraguaiacol due to its oxidation. The molar extinction coeffcient of tetraguaiacol was taken as 26.6 mM^−1^ cm^−1^. One unit of enzyme activity is defined as the formation of 1 µmol product of tetraguaiacol by the enzyme-catalyzing action per minute at 30°C.

### Protein content

Total soluble protein estimation was done by the Bradford reagent method (Bradford, 1976). The absorbance was recorded using a spectrophotometer at 595 nm (Bradford Protein assay, USA) and expressed in mg per gm of fresh weight. For the preparation of protein standard, 1 mg/ml bovine serum albumin was dissolved in distilled water and utilized as the stock solution.

### Statistical analysis

The analysis of variance and comparison of means were analyzed using HAU-OPSTAT software (http://hau.ernet.in/OPSTAT). Genetic parameters including the genotypic and phenotypic variance, genotypic and phenotypic coefficients of variance, heritability (broad sense), and expected genetic advance (GA) were performed using package variability 0.1.0 (Popat et al., 2020) through R software (ver. 4.2.1). The mean data for the recorded traits were used for calculating Pearson’s correlation coefcient by using ggplot 2 (R 2016) in R Studio (2021).

## Results

### Symptomatology and morphological and molecular identification of *Stemphylium vesicarium*

**Figure 1:**
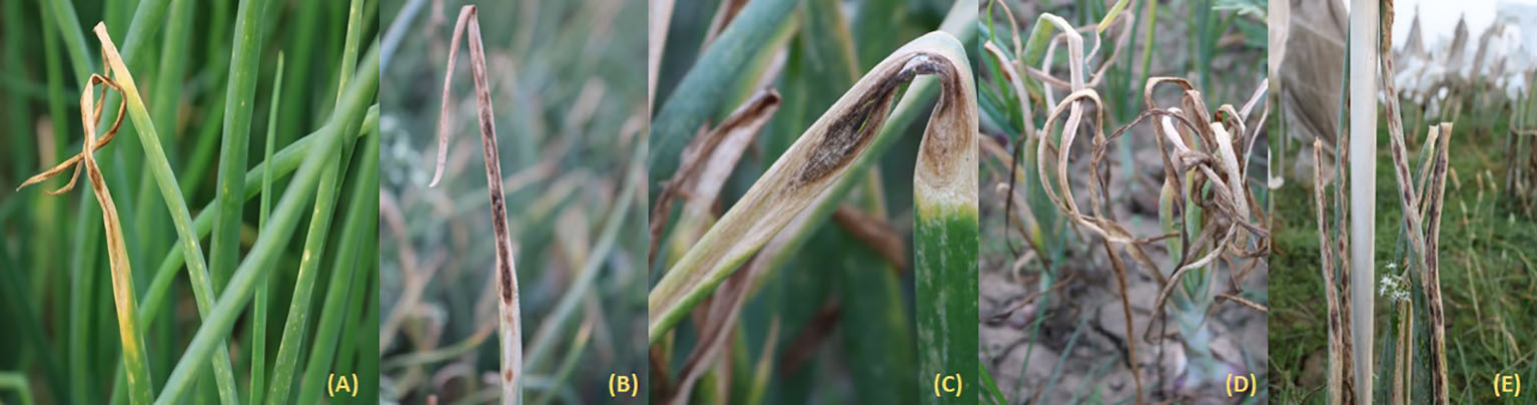
Symptoms of *Stemphylium* leaf blight. **(A)** Initial brown oval lesions formed on the leaf tip. **(B)** Lesions and their progression toward center. **(C)** Brown oval lesions formed in the center. **(D)** Complete drying of leaves. **(E)** Severe infection and breaking of floral stalk.

**Figure 2:**
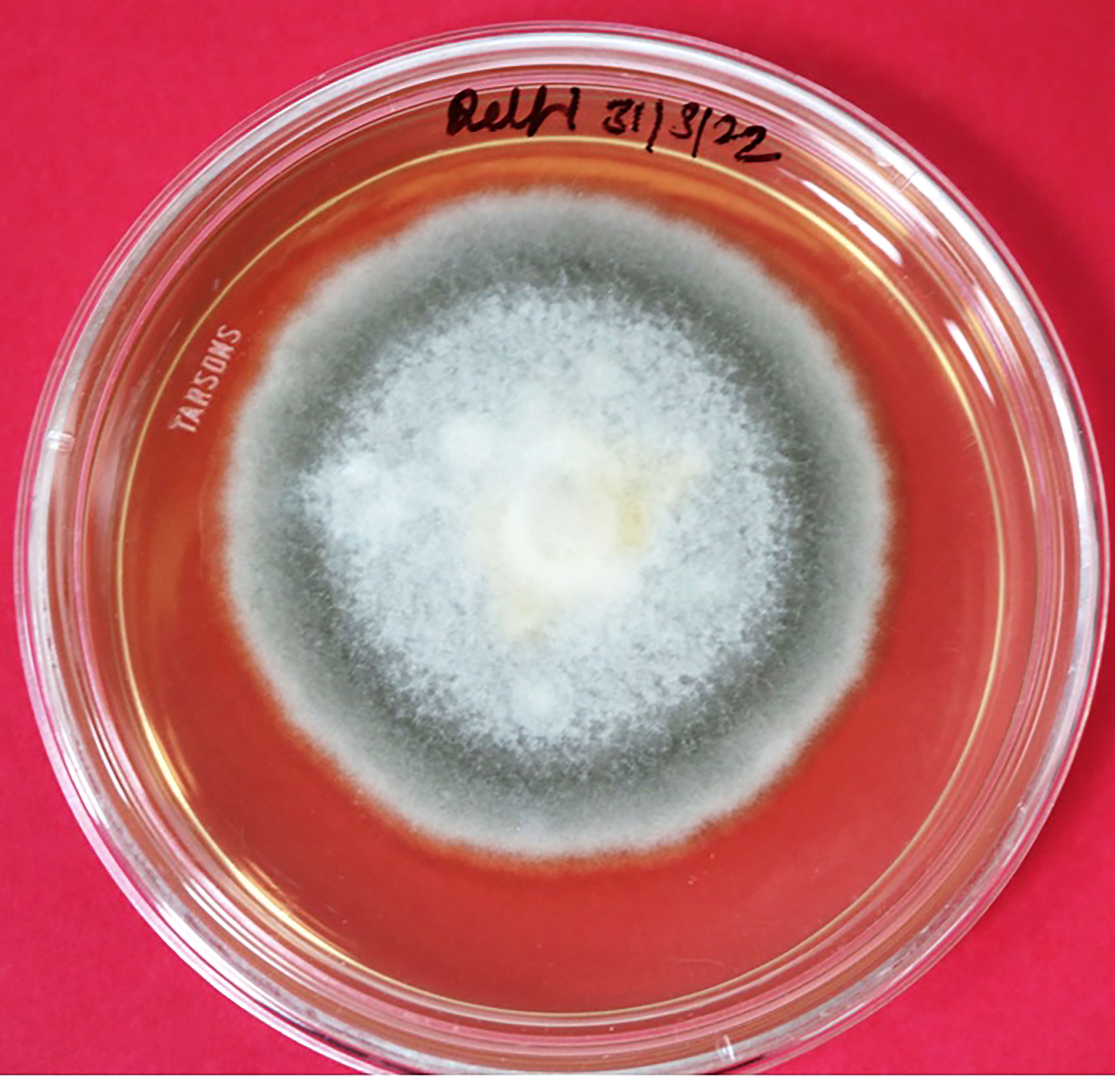
Isolation and purification of *Stemphylium vesicarium*. Whitish to light gray center, filiform, whitish margin, and septate mycelia observed on PDA media.

**Figure 3:**
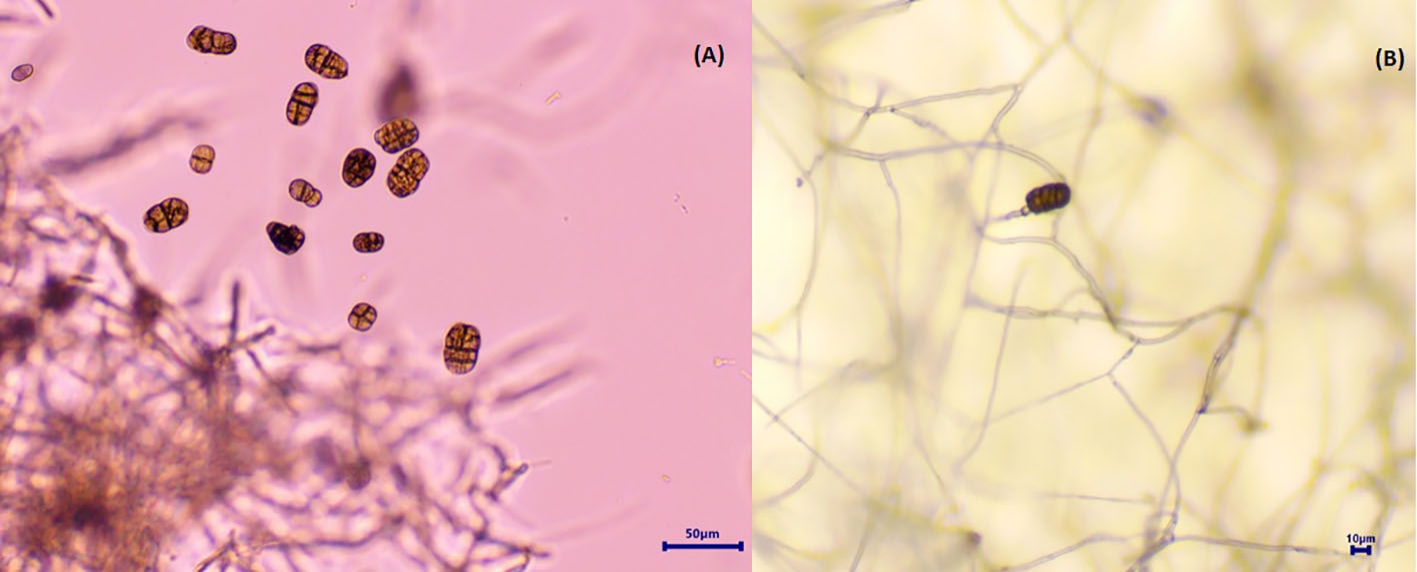
Spores of *Stemphylium vesicarium* on PDA media. **(A)** Conidia. **(B)** Conidia with conidiophores.

The infected onion leaves started to exhibit initial symptoms of SLB after 7–10 days of inoculation under field conditions. Initial symptoms included small tan to brown water-soaked, oval lesions on the tips and center of outer leaves. Necrosis started from the top and advanced downward on both sides of the lesions. Sometimes only tan necrosis occurred on the tip of leaves, which is not associated with any lesion formation and progressed toward the bottom of the leaves. In later stages, these symptoms advanced to dark-brown to black sporulation and were more evident on older plants. On outer leaves, black and purple target spot lesions with black sporulation were also evident occasionally (**Figure 1**). The isolate was identified as *Stemphylium vesicarium* on the basis of cultural characteristics and morphology of conidia and conidiophores. Whitish with light gray center, filiform, whitish margin colonies of *Stemphylium vesicarium* were observed on PDA medium (**Figure 2**). The observed conidia were light brown to brown in color, ovoid to oblong with zero to five transverse septa and one to five longitudinal septa, ranging 15–23 × 40–49 μm (**Figure 3**). Conidiophores were straight or occasionally one-branched with a swollen apex. The obtained ITS sequence showed 100% sequence homology with NCBI accessions of *S. vesicarium* (MZ452063). The generated sequence was submitted to the NCBI database (OP473965).

### Morphological and biochemical traits (stage I)

**Figure 4:**
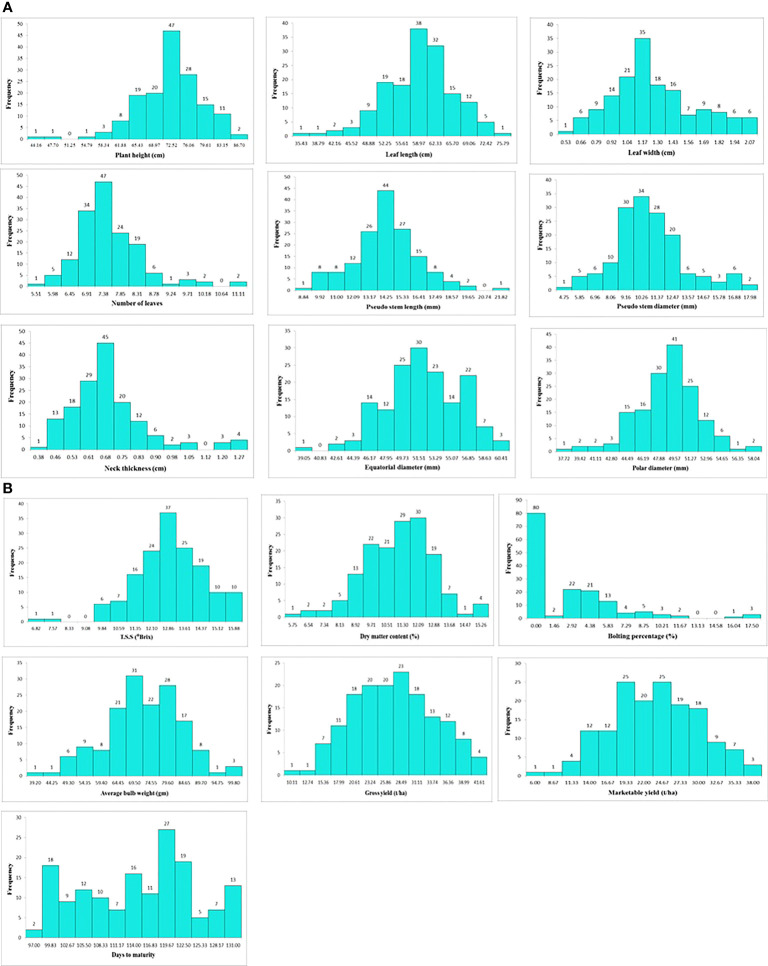
**(A)** Frequency distribution of onion genotypes for various morphological traits. **(B)** Frequency distribution of onion genotypes for morphological, biochemical, and yield traits.

Descriptive statistics of 16 morphological and biochemical traits indicated the presence of significant variation among onion genotypes under disease pressure (**Supplementary Table 2**). Approximately, a normal distribution was observed for most of the traits apart from bolting percentage and days to maturity (**Figures 4A, B**). Among various morphological traits, the plant height varied from 44.16 to 86.70 cm with a mean value of 70.31 cm (**Supplementary Table 1**). The plant height was recorded maximum in genotype SBO023 (86.70 cm) whereas the minimum was observed in SBO157 (44.16 cm). Similarly for leaf length, SBO023 (75.79 cm) recorded the highest value and minimum leaf length was recorded in SBO157 (35.43 cm). The leaf width was maximum in genotype SBO115 (2.07 cm) and minimum in SBO046 (0.53 cm) followed by SBO057 (0.54 cm). The number of leaves was maximum in SBO078 (11.11) and minimum in SBO083 (5.51). The genotype SBO091 (21.82 mm) followed by SBO109 (19.16 mm) exhibited the highest value for pseudostem length, whereas SBO06 (8.84 mm) had minimum pseudostem length. For trait pseudostem diameter, the maximum value was recorded for SBO038 (17.98 mm), whereas the minimum was observed in SBO132 (4.75 mm). Minimum neck thickness was recorded in SBO056 (0.38 cm), whereas the maximum was found in SBO080 (1.27 cm). The equatorial diameter of bulb was maximum in SBO148 (60.41 mm) and minimum in SBO091 (39.05 mm) with an average value of 51.20 mm. The genotype SBO148 (58.04 mm) was found have the highest value for polar diameter, whereas the minimum value was recorded for SBO131 (37.72 mm). A wide variability for average bulb weight was observed. The average bulb weight varied from 39.20 to 99.80 g with an average of 69.71 g. The highest average bulb weight was recorded in SBO148 (99.80 gm), whereas the lowest was observed in SBO038 (39.20 gm). The gross yield was recorded maximum in SBO020 (41.61 t/ha), whereas the lowest was observed in SBO076 (10.11 t/ha) followed by SBO116 (11.44 t/ha) and SBO138 (13.44 t/ha). The average marketable yield varied from 6.00 to 38.00 t/ha with an average of 22.22 (t/ha). The highest marketable yield was recorded in SBO071 (38.00 t/ha) followed by SBO120 (37.28 t/ha), and the least was recorded in SBO119 (6.00 t/ha). The minimum days to maturity were recorded for two genotypes SBO044 (97.00 days) and SBO073 (97.00 days), whereas the maximum days to maturity were observed for genotype SBO124 (131.00 days) followed by SBO116 (130.50). TSS ranged from 6.82 to 15.88 (°B) and showed a significant variation among genotypes. Maximum TSS was recorded in genotype SBO069 (15.88°B), whereas the minimum was found in genotype SBO145 (6.82°B). The average values of dry matter content was 10.76%. Maximum dry matter content was found in genotype SBO025 (15.26%) followed by SBO138 (14.96%), whereas the minimum dry matter content was recorded in genotype SBO090 (5.75%). Nearly 80 genotypes were present in a set of 157 genotypes which did not bolt at all, whereas the maximum bolting percentage was found in three genotypes SBO093, SBO118, and SBO119 (17.50%).

### Analysis of variance and estimates of genetic parameters

The analysis of variance was estimated for all the 17 traits which showed highly significant mean squares at P< 0.01 and 0.001, indicating variation among the 157 onion genotypes (**Table 2**). A wide range of values among the genotypes for all traits under consideration also indicates the presence of variation. Moreover, mean, standard errors, and coefficients of variation were also estimated which again confirmed considerable genetic variability among the genotypes. The phenotypic variance (σ^2^P) for all the traits was higher than the genotypic variance (σ^2^G). The phenotypic variance ranged from 0.03 (NT) to 128.94 (ABW), whereas the genotypic variance ranged from 0.03 (NT) to 126.93 (ABW). PCV and GCV values more than 20% are regarded as high, values ranging between 10% and 20% as medium, and values less than 10% as low (Deshmukh et al., 1986). GCV values ranged from 5.35% (Polar diameter) to 142.33% for the trait percent bolting. Similarly, the PCV values ranged from 8.28% for polar diameter to 150.03% for percent bolting. GCV values were lower than those of PCV values for all the traits. Traits such as leaf width, pseudostem diameter, neck thickness, percent bolting, gross yield marketable yield, and percent disease incidence were recorded with high GCV and PCV values. The highest GCV was observed for trait percent bolting (142.33%) followed by leaf width (29.57%) and PDI (28.70%). The medium GCV was recorded for traits such as leaf length, number of leaves, pseudostem length, total soluble solids, dry matter content, and average bulb yield. The plant height, equatorial diameter, polar diameter, and days to maturity were grouped under the traits having low GCV. The lowest GCV was recorded for polar diameter (5.35%) followed by equatorial diameter (7.11%) and days to maturity (8.52%). Similarly for traits such as percent bolting (150.03%), leaf width (29.68%), marketable yield (33.32%), gross yield (29.95%), PDI (29.14%), neck thickness (27.31%), and pseudostem diameter (24.38%), a high PCV was observed, whereas four traits, viz., plant height (9.63%), equatorial diameter (8.57%), polar diameter (8.28%), and days to maturity (8.57%), were recorded with the lowest PCV values. Medium PCV was recorded in five traits, namely, leaf length, number of leaves, pseudostem length, total soluble solids, dry matter content, and average bulb weight. The traits including polar diameter, percent bolting, gross yield, and marketable yield recorded a high difference among GCV and PCV values as compared with the rest of the traits which have a very small (<1) difference in their GCV and PCV values. Heritability values higher than 80% were considered as very high, values between 60% and 79% were moderately high, 40%–59% were medium, and values less than 40% were low (Singh, 2001). Heritability in a broad sense was also estimated for all traits, which ranged from 41.77% (polar diameter) to 99.51% (pseudostem width). Traits such as plant height, leaf length, leaf diameter, number of leaves, pseudostem length, pseudostem width, neck thickness, total soluble solids, dry matter content, bolting percentage, average bulb weight, days to maturity, and PDI were observed to have the highest heritability. Only one trait, i.e., equatorial diameter, was recorded with moderately high heritability. The remaining traits such as polar diameter, gross yield, and marketable yield were found to have medium heritability. Estimation of genetic advance for various traits ranged from 0.35% to 23.39% and was found highest for average bulb weight (23.39%). Moderate genetic advance was observed for traits such as days to maturity, PDI, plant height, and leaf length, whereas all the remaining traits were found to have low genetic advance.

**Table 2:** Genetic variability of 16 morphological and two biochemical traits of onion genotypes.

	Source of variation and mean squares			Estimation of genetic parameters				Performance					
Source of variation	Replication	Genotype	Mean	(σ^2^P)	(σ^2^G)	GCV	PCV	$h_{BS}^{2}$ (%)	GA	CV%	SEM (±)	CD 5%	CD 1%
df	1	156											
PH	0.46	90.56***	70.31	45.85	44.71	9.51	9.63	97.52	13.60	1.52	0.75	2.11	2.78
LL	0.63	96.01**	57.41	48.33	47.68	12.03	12.11	98.66	14.13	1.39	0.57	1.59	2.09
LW	0.002	0.25**	1.21	0.13	0.13	29.57	29.68	99.22	0.73	2.65	0.02	0.06	0.08
NOL	0.007	1.57***	7.39	0.79	0.78	12.10	12.22	98.00	1.80	1.73	0.09	0.25	0.33
PsL	0.11	9.34**	13.72	4.70	4.65	15.73	15.81	98.99	4.42	1.59	0.15	0.43	0.57
PsW	0.005	12.64**	10.33	6.34	6.31	24.32	24.38	99.51	5.16	1.71	0.12	0.35	0.46
NT	0.005	0.06***	0.66	0.03	0.03	26.66	27.31	95.33	0.35	5.92	0.03	0.08	0.10
ED	13.16	32.52***	51.20	15.82	6.61	7.11	8.57	68.76	3.42	6.02	1.73	4.84	6.39
PD	3.09	36.08**	48.01	14.88	6.58	5.35	8.28	41.77	3.00	6.32	1.61	4.49	5.93
T.S.S	0.56	5.06**	12.54	2.61	2.46	12.49	12.88	94.08	3.13	3.13	0.28	0.78	1.02
DM	0.02	6.06**	10.76	3.33	2.73	15.37	16.96	82.10	3.09	7.18	0.55	1.52	2.01
Blt	22.41	25.69***	2.45	13.52	12.17	142.33	150.03	90.01	6.82	47.42	0.82	2.30	3.03
ABW	0.009	260.70***	69.71	128.94	126.93	15.26	16.50	97.08	23.39	6.01	1.39	3.88	5.12
GY	2.20	93.90**	25.92	60.27	33.63	22.38	29.96	55.80	8.92	19.92	3.65	10.20	13.46
MY	3.16	85.52**	22.22	54.82	30.71	24.94	33.32	56.02	8.54	22.10	3.47	9.70	12.80
DTM	3.06	188.99**	113.73	95.00	93.99	8.52	8.57	98.94	19.86	0.88	0.71	1.98	2.62
PDI	38.67	178.55**	32.66	90.61	87.94	28.70	29.14	97.05	19.03	5.00	1.16	3.23	4.26

df, degree of freedom; GCV, genetic coefficient of variation; PCV, phenotypic coefficient of variation; ${\text{h}}_{\text{BS}}^{2}$ (%), heritability in a broad sense; GA, genetic advance; CV, coefficient of variance; SE, standard error; CD, critical difference; PDI, percent disease incidence (%); PH, plant height (cm); LL, leaf length (cm); LW, leaf width (cm); NoL, number of leaves; PsL, pseudostem length (mm); PsW, pseudostem width (mm); NT, neck thickness (cm); ED, equatorial diameter (mm); PD, polar diameter (mm); ABW, average bulb weight (gm); TSS, total soluble solids (°B); DM, dry matter (%); Blt, bolting percentage (%); DTM, days to maturity; MY, marketable yield (t/ha); GY, gross yield (t/ha); ns, non-significant, **, *** significant at p< 0.01, and 0.001, respectively.

### Screening the seed-to-bulb stage under field conditions (stage I)

**Figure 5:**
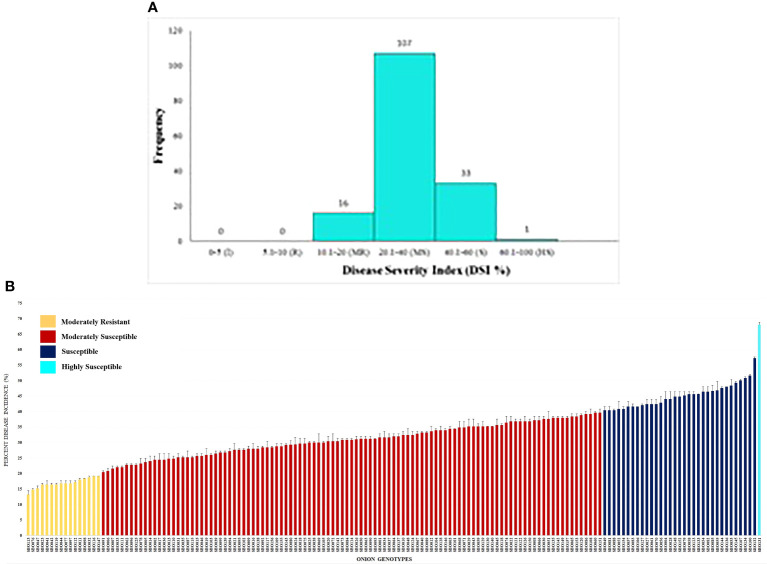
**(A)** Frequency distribution of onion genotypes based on percent disease incidence (PDI). **(B)** Reaction of onion genotypes based on percent disease incidence (PDI) to infection caused by *Stemphylium* leaf blight.

**Figure 6:**
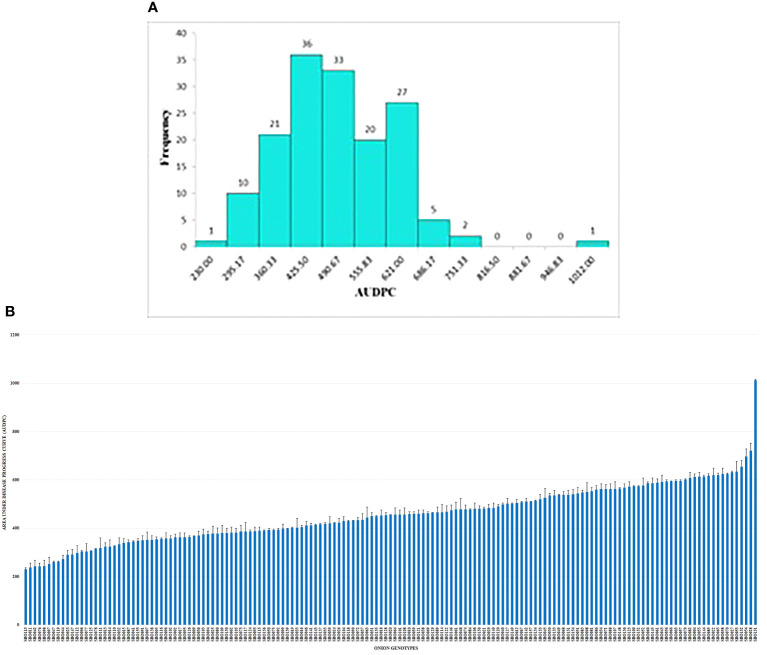
**(A)** Frequency distribution of onion genotypes based on the area under the disease progress curve (AUDPC). **(B)** Reaction of onion genotypes based on the area under the disease progress curve (AUDPC).

Field evaluation at 10, 20, and 30 DAI showed a wide phenotypic response. The mean values for the percent disease incidence of SLB among the 157 genotypes at 30 DAI ranged from 13.20% to 68.00% (**Supplementary Table 3**). The highest SLB incidence was observed in SBO131 (68.00%), and the lowest was recorded in SBO113 (13.20%). Only four categories of disease reaction existed among the 157 onion genotypes (**Figure 5A**). Out of 157 genotypes, 16 genotypes (10.20%) exhibited moderately resistant reactions whereas 107 genotypes (68.10%) were found to be moderately susceptible. Thirty-three genotypes (21.00%) were categorized as susceptible, and one (SBO131) genotype (0.70%) was found to be highly susceptible at 30 DAI (**Figure 5B**). None of the genotypes were found to be immune or resistant against SLB in this study. Out of 16 moderately resistant genotypes, three were open pollinating varieties (OPV), five were inbred lines, seven were breeding lines, and one was hybrid. The maximum numbers of moderately resistant (7), moderately susceptible (36), and susceptible (13) genotypes were found among breeding lines. Among all genotypes belonging to OPVs, breeding lines, inbred lines, and hybrids, only one genotype belonging to the OPV group exhibited a highly susceptible reaction (**Table 3**). The area under the disease progress curve ranged from 230.00 to 1012.00 and was recorded minimum in SBO113 (230.00) and maximum in SBO131 (1012.00) (**Figures 6A, B**). The results for the lowest and highest mean values of PDI and AUDPC were similar, but they were not identical in ranking of the genotypes, e.g., ‘SBO076’ had a PDI of 14.80 and was ranked in second place on PDI basis but was ranked fourth based on the AUDPC scale (**Supplementary Table 3**).

**Table 3:** Percent disease incidence (PDI) categories of 157 onion genotypes against *Stemphylium vesicarium*.

Category	Genotypes	PDI	DR	Genotype name	No. of genotypes
OPV	45	<5.0	I	–	–
		5.1-10	R	–	–
		10.1-20	MR	SBO098, SBO023, SBO032	3
		20-40	MS	SBO013, SBO020, SBO021, SBO022, SBO024, SBO025. SBO026, SBO029, SBO030, SBO031, SBO033, SBO034, SBO035, SBO036, SBO037, SBO089, SBO090, SBO093, SBO094, SBO095, SBO096, SBO099, SBO101, SBO102, SBO103, SBO104, SBO105, SBO130, SBO137, SBO138, SBO139, SBO142, SBO143, SBO156	34
		40.1-60	S	SBO27, SBO28, SBO100, SBO106, SBO133, SBO134, SBO144	7
		>60.0	HS	SBO131	1
Inbreds	38	<5.0	I	–	–
		5.1-10	R	–	–
		10.1-20.0	MR	SBO047, SBO076, SBO097, SBO116, SBO119	5
		20.1-40.0	MS	SBO002, SBO003, SBO005, SBO007, SBO008, SBO009, SBO010, SBO016, SBO018, SBO039, SBO040, SBO043, SBO045, SBO063, SBO066, SBO078, SBO080, SBO084, SBO086, SBO087, SBO109, SBO111, SBO114, SBO118, SBO129	25
		40.1-60	S	SBO054, SBO079, SBO081, SBO082, SBO083, SBO085, SBO107, SBO154	8
		>60	HS	–	–
Breeding lines	56	<5	I		–
		5.1-10	R		–
		10.1-20	MR	SBO011, SBO041, SBO042, SBO044, SBO077, SBO112, SBO113	7
		20-40	MS	SBO001, SBO012, SBO006, SBO014, SBO015, SBO017, SBO019, SBO038, SBO046, SBO048, SBO050, SBO051, SBO059, SBO060, SBO062, SBO064, SBO065, SBO067, SBO068, SBO069, SBO071, SBO074, SBO075, SBO091, SBO092, SBO108, SBO110, SBO115, SBO117, SBO124, SBO125, SBO126, SBO128, SBO150, SBO151, SBO153	36
		40.1-60	S	SBO004, SBO049, SBO052, SBO053, SBO055, SBO056, SBO057, SBO058, SBO061, SBO070, SBO088, SBO127, SBO152	13
		>60	HS	–	–
Hybrids		<5	I	–	–
		5.1-10	R	–	–
		10.1-20	MR	SBO147	1
		20-40	MS	SBO072, SBO073, SBO120, SBO121, SBO122, SBO135, SBO140, SBO141, SBO1146, SBO149, SBO155, SBO157	12
		40.1-60	S	SBO123, SBO132, SBO136, SBO145, SBO148	5
		>60	HS	–	–

OPV, open pollinated varieties; DR, disease rating.

### Pearson’s correlation coefficient under stage I

Concerning the phenotypic correlation coefficient, the results revealed that there was a significant positive correlation present between PDI and AUDPC (p< 0.0001). On the other hand, PDI was found to be significantly negatively correlated with leaf length (r = -0.20*), number of leaves (r = -0.17*), pseudostem width (r = -0.31***), neck thickness (r = -0.21**), and dry matter (r = -0.29***) (**Table 4**). The correlation between plant height and leaf length was highly significant and positive (r = 0.94***). Traits like leaf width, number of leaves, neck thickness, pseudostem length, pseudostem width, equatorial diameter, polar diameter, gross yield, and marketable yield were also found to be significantly positively correlated with plant height. A significant but very low positive correlation was observed among leaf length and traits such as leaf width, number of leaves, pseudostem width, neck thickness, polar diameter, average bulb weight, marketable yield, and gross yield. Leaf width was found to be significantly positively correlated with number of leaves, pseudostem length, pseudostem width, neck thickness, average bulb weight, and days to maturity. A significant positive correlation was recorded among number of leaves and traits like pseudostem width, neck thickness, days to maturity, and gross yield. Pseudostem length was found to be significantly negatively correlated with equatorial diameter (r = -0.17*), whereas pseudostem width was observed to have a significant positive correlation with neck thickness and days to maturity. Neck thickness and days to maturity were recorded to have a significant positive correlation among them. Equatorial diameter and polar diameter of bulb were significantly positively correlated with average bulb weight, gross yield, and marketable yield. A negative correlation was also found among polar diameter and TSS (r = -0.23**). The average bulb weight showed a significant positive correlation with traits like marketable yield (r = 0.57**) and gross yield (r = 0.56**), whereas it was found to be negatively correlated with TSS (r = -0.21**). A significant positive correlation was observed among TSS and dry matter content (r = 0.35**), whereas TSS was found to be negatively correlated with marketable yield (r = -0.23**) and gross yield (r = -0.30**). The bolting percentage was also recorded as significantly negatively correlated with marketable yield (r = -0.27**) and gross yield (r = -0.28**).

**Table 4:** Pearson’s correlation coefficient of PDI with morphological and biochemical traits.

	PDI	AUDPC	PH	LL	LW	NoL	PsL	PsW	NT	ED	PD	ABW	T.S.S	DM	Blt	DTM	MY	GY
**PDI**	1	0.91**	-0.13	-0.20*	0.05	-0.17*	0.05	-0.31***	-0.21**	0.12	-0.09	0.09	-0.01	-0.29***	0.09	-0.07	0.02	0.02
**AUDPC**		1	-0.12	-0.16*	-0.06	-0.17*	0.01	-0.26**	-0.20*	0.09	-0.11	0.07	0.04	-0.31***	0.05	-0.06	0.01	0.01
**PH**			1	0.94***	0.24**	0.38***	0.17*	0.21***	0.19*	0.07	0.20*	0.22**	-0.05	0.03	-0.06	0.04	0.23**	0.24**
**LL**				1	0.23**	0.37***	0.08	0.25**	0.22**	0.04	0.17*	0.17*	-0.07	0.04	-0.07	0.02	0.16*	0.18*
**LW**					1	0.22**	0.23**	0.38***	0.40***	0.05	-0.04	0.16*	-0.06	0.08	0.11	0.22**	0.14	0.14
**NoL**						1	0.07	0.32***	0.35***	0.03	0.00	0.10	-0.05	0.01	0.10	0.30***	0.15	0.22**
**PsL**							1	-0.09	-0.05	-0.17*	-0.05	-0.08	-0.14	-0.08	0.11	0.01	0.05	0.03
**PsW**								1	0.82***	0.05	-0.11	0.16*	0.05	0.14	-0.03	0.29***	0.13	0.15
**NT**									1	0.10	-0.03	0.10	-0.04	0.03	0.05	0.28***	0.07	0.10
**ED**										1	0.41***	0.52***	-0.07	0.00	-0.05	-0.01	0.26**	0.26**
**PD**											1	0.43***	-0.23**	-0.13	-0.17*	-0.13	0.35***	0.39***
**ABW**												1	-0.21**	0.00	-0.07	0.06	0.57***	0.56***
**T.S.S**													1	0.35**	0.10	-0.02	-0.23**	-0.30***
**DM**														1	-0.04	0.01	0.06	0.00
**Blt**															1	-0.03	-0.27***	-0.28***
**DTM**																1	0.03	0.04
**MY**																	1	0.97***
**GY**																		1

PDI, percent disease incidence (%); AUDPC, area under disease progress curve; PH, plant height (cm); LL, leaf length (cm); LW, leaf width (cm); NoL, number of leaves; PsL, pseudostem length (mm); PsW, pseudostem width (mm); NT, neck thickness (cm); ED, equatorial diameter (mm); PD, polar diameter (mm); ABW, average bulb weight (gm); TSS, total soluble solids (°B); DM, dry matter (%); Blt, bolting percentage (%); DTM, days to maturity; MY, marketable yield (t/ha); GY, gross yield (t/ha); ns, non-significant, *, **, *** significant at p< 0.05, 0.01, and 0.001, respectively.

### Screening the bulb-to-seed stage under controlled conditions (stage II)

**Figure 7:**
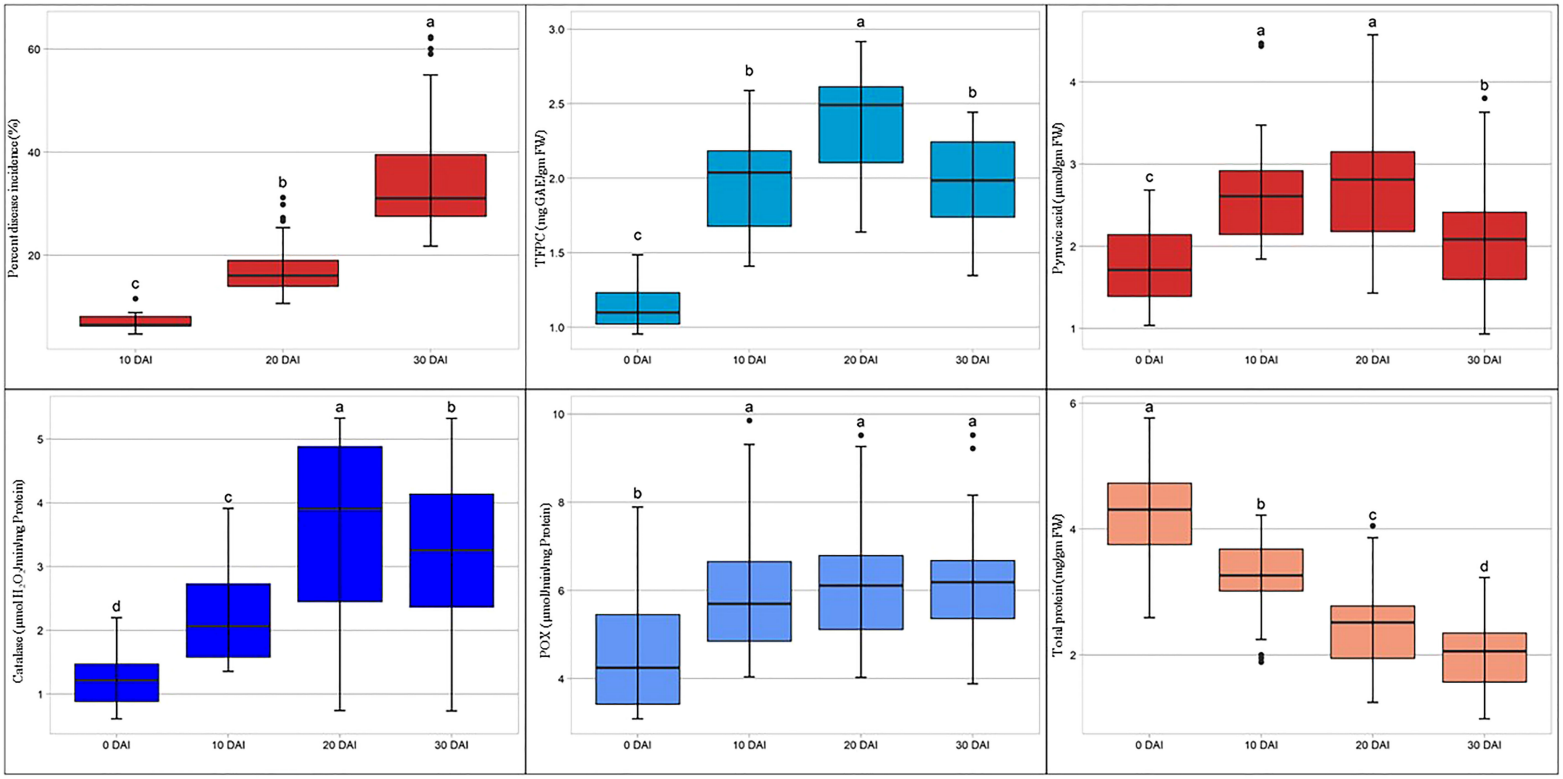
Box plot representation for mean activity of biochemical compounds, before inoculation and after different days intervals.

For screening and biochemical characterization at different intervals under controlled conditions, 20 genotypes were selected. Genotypes were selected based on their phenotypic response to SLB under artificial inoculated field conditions. The results pertaining to the screening and biochemical characterization at different day intervals under controlled conditions are presented in **Table 5**. The leaves showed initial symptoms of SLB after 7 days, and the first observation was recorded at 10th DAI. The lowest PDI was recorded in genotype SBO113 (4.73%), whereas the highest was observed in SBO131 (11.56%) at 10 DAI. Similarly at 20 DAI, genotype SBO113 (10.66%) followed by SBO097 (11.15%) and SBO116 (13.28%) showed minimum values for PDI, whereas the maximum PDI was recorded in SBO131 (30.51%) followed by SBO100 (25.33%). At 30 DAI, genotypes SBO113 (22.86%) followed by SBO097 (25.12%) exhibited the lowest PDI values, whereas SBO131 (62.22%) followed by SBO132 (59.54%) was recorded with the highest PDI. Biochemical compounds were also estimated before inoculation and after inoculation at the 10-day interval up to 30 days (**Table 5**). The accumulation of different biochemical compounds showed an increasing trend and was found highest at 20 days except protein content (**Figure 7**). Protein content was highest at the initial stage, but it kept on decreasing after inoculation. Before inoculation, the total foliar phenol ranged from 0.96 to 1.45 mg GAE/gm fw. The maximum accumulation of total foliar phenol content was recorded in genotype SBO023 (1.45), and the minimum was observed in SBO112 (0.96). Pyruvic acid in leaves was estimated highest in SBO116 (2.68 μmol/gm fw), whereas the lowest was recorded in SBO112 (1.05 μmol/gm fw). The catalase activity was found to be maximum in SBO113 (2.18 μmol H_2_O_2_/min/mg Prot.), whereas the minimum was observed in SBO134 (0.61 μmol H_2_O_2_/min/mg Prot.). Peroxidase activity was recorded maximum in genotype SBO097 (7.76 μmol/min/mg Prot.), and minimum activity was observed in SBO077 (3.21 μmol/min/mg Prot.). The maximum total protein level in leaves was recorded in genotype SBO041 (5.60 mg/gm fw). After 10 days of inoculation, the maximum total foliar phenol was observed in SBO041, and the minimum was recorded in SBO100. The genotype SBO097 showed the highest accumulation of pyruvic acid (4.45) and peroxidase (9.58). Accumulation of pyruvic acid and peroxidase activity was found to be minimum in SBO132. Catalase activity and total protein were recorded maximum in SBO113, whereas the lowest values for catalase activity were recorded in SBO131, and for total protein, it was observed in SBO134. The genotype SBO113 showed the highest accumulation for total foliar phenol, catalase, and protein, whereas genotype SBO131 recorded the lowest accumulation of the same at 20 days. Also, concentrations of pyruvic acid and peroxidase were found maximum in genotype SBO097, whereas the minimum was observed in SBO132. Similarly at 30 DAI, genotype SBO113 was found to have the highest concentration of total foliar phenol, catalase, and total protein. The pyruvic acid and peroxidase accumulation was also recorded maximum in SBO097. The genotype SBO131 recorded the lowest concentration of total foliar phenol, catalase, and peroxidase, whereas genotypes SBO132 and SBO134 were observed with the lowest accumulation of pyruvic acid and total protein, respectively.

**Table 5:** Mean activity of biochemical compounds, before inoculation and after inoculation, at different day intervals.

		Before inoculation							10 days after inoculation					
S. No.	Genotypes	TFPC	PAC		CAT		POX	PC	PDI	TFPC	PAC	CAT	POX	PC
1.	**SBO011**	1.13^ef^	1.48^jk^		1.40^efg^		5.55^d^	3.97^ef^	6.78^cdef^	2.01^h^	2.91^de^	1.97^fg^	5.86^efg^	3.06^fg^
2.	**SBO023**	1.45^a^	2.08^e^		1.78^bc^		6.51^c^	3.84^fg^	6.21^defg^	2.16^ef^	2.75^fg^	2.74^c^	7.51^bc^	3.31^def^
3.	**SBO032**	1.28^b^	1.56^i^		0.77l^m^		5.53^d^	4.80^bc^	6.38^cdefg^	2.05^gh^	2.55^h^	1.91^g^	6.78^cd^	3.46^cde^
4.	**SBO041**	1.22^bcd^	2.39^c^		1.66^cd^		3.40^g^	5.60^a^	6.43^cdefg^	2.56^a^	2.93^d^	3.47^b^	5.57^fgh^	3.98^ab^
5.	**SBO042**	1.16^cde^	2.32^d^		1.32^fgh^		5.16^e^	4.50^bcd^	5.69^efg^	2.15^efg^	2.67^g^	2.08^efg^	6.65^de^	3.58^cd^
6.	**SBO044**	1.28^b^	1.22^m^		1.25^ghi^		5.21^de^	3.85^fg^	7.32^bcde^	1.47^lm^	2.16^jk^	2.10^ef^	5.48^fghi^	3.23^def^
7.	**SBO047**	1.00^hi^	1.71^h^		1.52^de^		4.22^f^	4.59^bcd^	6.39^cdefg^	2.21^de^	2.74^fg^	2.80^c^	5.29^ghi^	3.95^ab^
8.	**SBO076**	1.05^gh^	1.75^gh^		0.86^kl^		4.06^f^	4.41^cd^	6.59^cdef^	2.42^b^	2.35^i^	1.56^hi^	4.61^ij^	3.07^fg^
9.	**SBO077**	1.15^de^	1.94^f^		1.20^hi^		3.21^g^	4.01^ef^	6.24^defg^	2.00^h^	3.43^b^	2.19^e^	5.36^ghi^	3.12^efg^
10.	**SBO097**	1.08^fg^	2.51^b^		1.09^ij^		7.76^a^	3.59^fg^	6.28^cdefg^	2.36^bc^	4.45^a^	2.85^c^	9.58^a^	2.96^fg^
11.	**SBO098**	0.96^i^	1.81^g^		1.45^ef^		7.22^b^	3.43^g^	6.47^cdef^	1.536^l^	2.82^ef^	2.41^d^	8.01^b^	2.12^h^
12.	**SBO100**	0.99^hi^	1.29^l^		0.75^lm^		4.44^f^	4.83^bc^	7.98^bc^	1.41^m^	1.94^m^	1.44^hi^	4.79^hij^	3.53^cd^
13.	**SBO112**	0.96^i^	1.05^n^		0.76^lm^		3.28^g^	5.33^a^	7.88^bcd^	2.09^fgh^	2.22^j^	1.60^h^	5.63^fgh^	3.96^ab^
14.	**SBO113**	1.22^bcd^	2.27^d^		2.18^a^		5.38^de^	4.76^bc^	4.73^g^	2.29^cd^	3.46^b^	3.86^a^	6.27^def^	4.13^a^
15.	**SBO116**	1.28^b^	2.68^a^		1.92^b^		4.17^f^	4.49^bcd^	5.20^fg^	2.11^efg^	3.11^c^	2.84^c^	6.75^cd^	2.99^fg^
16.	**SBO119**	1.07^fg^	1.43^k^		0.99^jk^		3.30^g^	4.85^b^	6.42^cdefg^	1.83^i^	2.05^l^	1.45^hi^	5.37^ghi^	3.75^bc^
17.	**SBO131**	1.01^ghi^	1.74^h^		0.87^kl^		4.26^f^	3.76^fg^	11.56^a^	1.53^l^	2.36^i^	1.37^i^	4.62^ij^	2.85^g^
18.	**SBO132**	1.03^ghi^	1.54^ij^		1.19^hi^		3.32^g^	3.53^g^	8.71^b^	1.63^k^	1.85^1m^	2.09^ef^	4.25^j^	3.13^efg^
19.	**SBO134**	0.97^i^	1.06^n^		0.61^m^		3.39^g^	2.59^h^	8.82^b^	1.77^ij^	2.09^kl^	1.50^hi^	4.65^ij^	1.91^h^
20.	**SBO145**	1.22^bc^	1.07^n^		1.10^ij^		4.14^f^	4.30^de^	8.88^b^	1.70^jk^	2.09^kl^	1.59^h^	6.58^de^	3.15^efg^
	**SE(m) ±**	0.01	0.02		0.05		0.11	0.12	0.50	0.02	0.03	0.05	0.26	0.11
	**SE(d) ±**	0.02	0.03		0.07		0.16	0.17	0.71	0.04	0.04	0.07	0.37	0.15
	**CV (%)**	2.21	2.14		6.16		3.53	4.20	10.15	2.00	1.80	3.56	6.20	4.85
		**20 days after inoculation**							**30 days after inoculation**					
**S. No.**	**Genotypes**	**PDI**	**TFPC**	**PAC**		**CAT**	**POX**	**PC**	**PDI**	**TFPC**	**PAC**	**CAT**	**POX**	**PC**
1.	**SBO011**	17.23^efg^	2.25^f^	3.04^ef^		4.52^ab^	5.43^ghij^	2.25^h^	34.15^e^	1.86^fg^	2.22^de^	4.42^b^	5.26^h^	1.81^e^
2.	**SBO023**	13.99^hi^	2.63^bc^	2.92^gh^		5.21^a^	6.26^defg^	2.65^def^	28.00^gh^	2.23^bc^	2.29^d^	3.93^cd^	5.94^ef^	2.36^cd^
3.	**SBO032**	16.00^efgh^	2.50^de^	3.12^e^		3.96^abcd^	6.92^cd^	2.62^def^	32.02^f^	1.91^f^	1.94^g^	3.85^cd^	6.55^d^	2.18^d^
4.	**SBO041**	14.33^hi^	2.82^a^	3.45^c^		4.90^a^	6.56^de^	3.26^b^	27.03^gh^	2.28^b^	2.71^c^	3.48^ef^	6.23^de^	2.86^b^
5.	**SBO042**	15.02^ghi^	2.77^a^	2.69^i^		2.82^cdef^	7.96^b^	2.82^cde^	28.44^g^	2.28^b^	2.11^ef^	2.38^i^	7.21^c^	2.35^cd^
6.	**SBO044**	16.11^efgh^	2.20^fg^	2.69^i^		4.4^ab^	6.07^defgh^	2.55^efg^	30.42^f^	1.85^fg^	2.08^f^	4.38^b^	5.72^fg^	2.19^d^
7.	**SBO047**	13.65^1i^	2.64^b^	3.00^fg^		5.14^a^	6.50^def^	3.00^c^	26.66^hi^	2.31^b^	2.25^d^	2.82^h^	6.35^de^	2.50^c^
8.	**SBO076**	14.47^hi^	2.58^bcd^	2.58^j^		3.13^bcdef^	5.95^efghi^	2.89^cd^	28.31^gh^	2.21^bcd^	2.05^fg^	2.88^h^	5.78^fg^	2.38^cd^
9.	**SBO077**	17.33^ef^	2.42^e^	2.89^h^		2.93^bcdef^	5.15^ijk^	2.46^fgh^	32.09^f^	1.93^f^	1.32^i^	2.96^gh^	4.86^i^	1.70^ef^
10.	**SBO097**	11.15^j^	2.51^de^	4.56^a^		4.13^abc^	9.39^a^	2.66^def^	25.12^i^	2.16^cd^	3.71^a^	4.04^c^	8.61^a^	2.21^d^
11.	**SBO098**	17.85^e^	1.71^i^	2.73^i^		3.20^bcdef^	7.41^bc^	1.88^i^	35.27^e^	1.468^i^	2.04f^g^	3.25^fg^	7.17^c^	1.38^gh^
12.	**SBO100**	25.33^bc^	1.71^i^	1.77^n^		2.03^f^	4.72^jkl^	1.40^j^	54.68^c^	1.49^i^	1.11^j^	0.88^j^	4.26^j^	1.37^efg^
13.	**SBO112**	16.12^efgh^	2.59^bcd^	2.00^l^		3.70^abcde^	5.66^fghi^	2.58^efg^	32.12^f^	2.05^e^	1.69^h^	3.64^e^	5.39^gh^	2.16^d^
14.	**SBO113**	10.66^j^	2.87^a^	3.60^b^		5.27^a^	6.42^def^	3.95^a^	22.86^j^	2.41^a^	3.131^b^	4.77^a^	6.30^de^	3.14^a^
15.	**SBO116**	13.28^i^	2.60^bcd^	3.21^d^		5.14^a^	7.93^b^	2.77^cde^	25.28^i^	2.24^bc^	2.69^c^	2.91^h^	8.09^b^	2.11^d^
16.	**SBO119**	15.36^fghi^	2.53^cd^	3.22^d^		3.95^abcd^	6.10^defgh^	2.31^gh^	28.49^g^	2.12^de^	2.65^c^	4.01^c^	6.07^ef^	1.8^e^
17.	**SBO131**	30.51^a^	1.66^i^	2.24^k^		1.784^f^	4.36^kl^	1.87^i^	62.22^a^	1.36^i^	1.73^h^	0.76^j^	3.99^j^	1.43^fg^
18.	**SBO132**	27.23^b^	1.70^i^	1.44°		2.14^ef^	4.12^l^	1.96^i^	59.54^b^	1.42^i^	0.95^k^	2.15^i^	3.99^j^	1.46^fg^
19.	**SBO134**	24.66^c^	2.13^g^	1.93^lm^		2.29^ef^	4.33^kl^	1.5^j^	51.84^d^	1.77^gh^	1.22^ij^	2.26^i^	4.23^j^	1.12^h^
20.	**SBO145**	20.66^d^	2.02^h^	1.90^m^		2.38^def^	5.27^hij^	1.80^i^	51.20^d^	1.70^h^	1.23^ij^	2.24^i^	5.01^hi^	1.63^efg^
	**SE(m)±**	0.67	0.03	0.02		0.47	0.26	0.08	0.57	0.03	0.04	0.10	0.12	0.08
	**SE(d)±**	0.94	0.04	0.04		0.66	0.37	0.12	0.81	0.05	0.05	0.15	0.18	0.12
	**CV (%)**	5.40	1.89	1.44		18.20	6.03	4.99	2.26	2.54	2.79	4.82	3.12	5.91

PDI, percent disease incidence (%); TFPC, total foliar phenol content (mg GAE/gm fresh weight); PAC, pyruvic acid content (μmol/gm fresh weight); CAT, catalase (μmol H_2_O_2_/min/mg protein); POX, guaiacol peroxidase (μmol/min/mg protein); PC, protein content (mg/gm fresh weight). Mean values followed by the same letter (superscipt) in the column do not differ statistically from each other by the Duncan’s Multiple-Range Test (DMRT) at the 5% probability level.

### Correlation studies between PDI and biochemical parameters (stage II)

**Figure 8:**
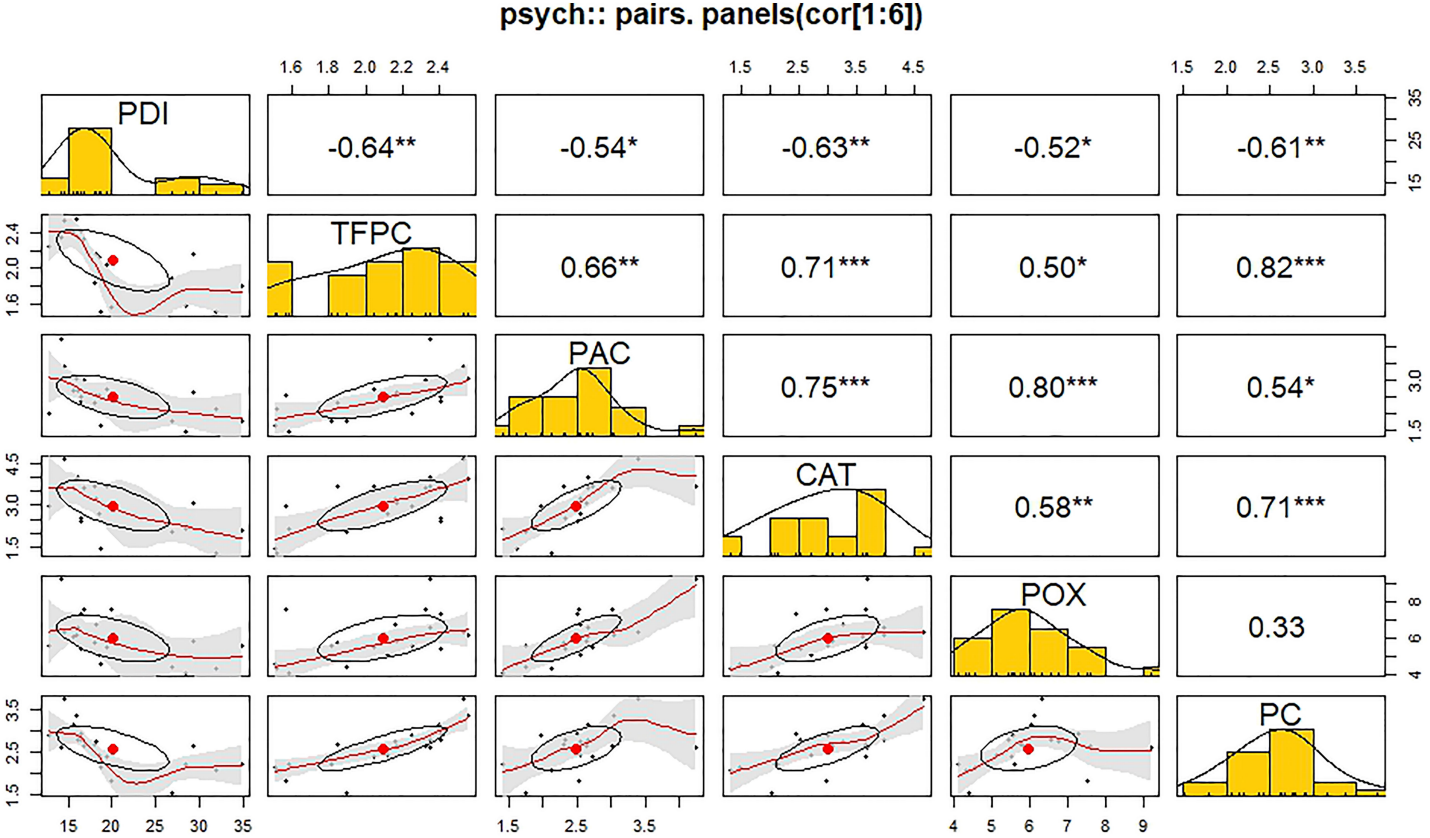
Correlation matrix (Pearson’s method) of different biochemical parameters in response to *Stemphylium vesicarium* incidence.

The Pearson correlation coefficient revealed a significant negative correlation between PDI and biochemical parameters during stage II (**Figure 8**). A significant negative correlation was found between PDI and TFPC (r = -0.64**). On the other hand, a significant positive correlation was found among the biochemical parameters. TFPC exhibited a highly significant correlation with protein content (r = 0.82***) and catalase (r = 0.71***) in the positive direction. Pyruvic acid content was found to have a highly significant positive correlation with guaiacol peroxidase (r = 0.80**) and catalase (r = 0.75***). Catalase was found to be significantly positively correlated with guaiacol peroxidase (r = 0.58**) and protein content (r = 0.71**). The correlation between guaiacol peroxidase and protein content was found to be non-significant in this study.

### Change in PDI between two stages (seed to bulb versus bulb to seed)

**Figure 9:**
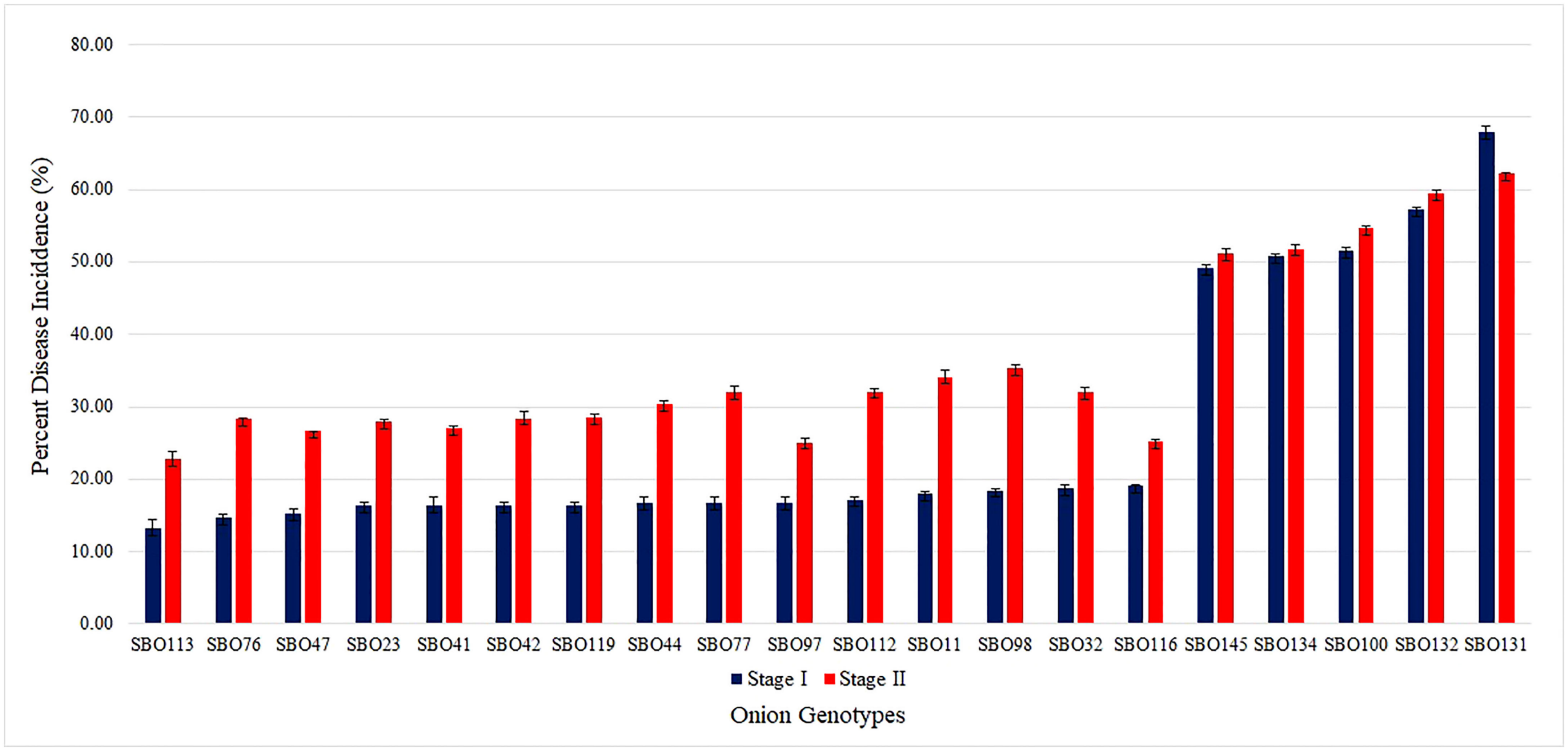
Comparison among percent disease incidence (PDI) of selected genotypes under stage I and stage II.

During the seed-to-bulb stage (stage I), under artificially inoculated field conditions, 15 genotypes were selected as moderately resistant and the PDI ranged from 13.20 to 19.20 whereas five genotypes with a PDI range of 49.20–68.00 were selected. These genotypes, in bulb form, were again screened under controlled conditions to assess their resistance ability under the bulb-to-seed stage. It was observed that all the genotypes categorized as moderately resistant (MR) in the first stage were categorized as susceptible in the bulb-to-seed stage. PDI ranged from 22.86 to 35.27 in the genotypes categorized as MR earlier. In the susceptible category, the PDI range was 49.20–68.00 in the seed stage, and in the bulb stage, the PDI ranged from 51.20 to 62.22 (**Figure 9**).

## Discussion

*Stemphylium* leaf blight has emerged as a serious constraint for onion production across major onion-growing areas throughout the world (Hay et al., 2021). The disease not only poses a significant threat to winter/*rabi* bulb crop but also has a negative impact on the onion seed yield too. *Stemphylium* leaf blight is also known to cause premature leaf senescence, which makes harvested onion crop more prone to postharvest losses (Paibomesai et al., 2012). Since no effective resistance exists in commercial varieties or cultivars, the disease has been extensively managed through fungicide application. Management of SLB through frequent fungicide applications has been associated with several major concerns such as resistance development among pathogens for a particular fungicide or a group of fungicides (Brown, 2006; Hay et al., 2021), environmental pollution, and human health hazard (Kumar, 2007). The evident impacts of these hazardous chemicals on the bee population could lead to a drastic reduction in pollination potential of honey bees for hybrid seed production in onion (Gillespie et al., 2014; McArt et al., 2017). In the current study, firstly a pure culture of *Stemphylium vesicarium* was isolated and artificial field inoculation was carried out on onion plants. Small tan to brown water-soaked, oval lesions were observed, and leaf samples were collected for microscopic and molecular identification. The microscopic observation revealed the presence of ovoid to oblong, brown color conidia, and these morphological observations coincided with the previous reports (Dangi et al., 2019; Gedefaw et al., 2019; Hassan et al., 2020). Also, the amplified product of the internal transcribed spacer (ITS) sequence, identified using NCBI BLAST, had 100% similarity with previously submitted sequences. *Stemphylium vesicarium* belongs to the *Pleosporaceae* family which is a very large group of fungi, and *Stemphylium* has a broad host range.

For various morphological traits including yield, significant variations have been reported (Mallor Giménez et al., 2011; Solanki et al., 2015; Dangi et al., 2019). In the present experiment also, different morphological traits including yield showed a significant variation among genotypes. The genotypes SBO071 (38 t/ha) followed by SBO120 (37.27 t/ha) recorded the highest marketable yield. Both SBO071 and SBO120 are F_1_ hybrids. F_1_ hybrids yield higher than open pollinated varieties and are known to be moderately resistant to insect pest and diseases due to their heterotic potential. Despite the higher *Stemphylium* leaf blight incidence, these genotypes gave superior yield performance and also possessed high total soluble solids and dry matter content. Hence, they can be recommended for cultivation in *Stemphylium* leaf blight-affected areas until new sources of resistance are identified.

Genotypic coefficients of variance coupled with heritability estimates provide better understanding about the variability present among genotypes and amount of advance to be expected from selection for various quantitative traits under consideration (Burton and Devane, 1953; Khosa and Dhatt, 2013; Dangi et al., 2018). GCV values were less than the PCV values for all the traits, which shows that the environment has a significant role in trait expression. Higher values for PCV than the value for GCV have been reported earlier (Khosa and Dhatt, 2013; Arya et al., 2017; Santra et al., 2017; Dangi et al., 2018). In agreement with our findings, the highest GCV and PCV were reported for leaf width, pseudostem diameter, neck thickness, percent bolting, gross yield marketable yield, and percent disease incidence (Khosa and Dhatt, 2013; Solanki et al., 2015; Dangi et al., 2018), whereas medium GCV and PCV were observed for traits such as leaf length and number of leaves (Khosa and Dhatt, 2013; Dangi et al., 2018), pseudostem length, total soluble solids, dry matter content, and average bulb yield (Awale et al., 2011; Solanki et al., 2015; Santra et al., 2017). The lowest GCV and PCV for plant height, days to maturity, equatorial diameter, and polar diameter (Khosa and Dhatt, 2013; Chattopadhyay et al., 2013; Santra et al., 2017) have been reported and are in agreement with our results. Population under study, environment, and the method used are the three major factors required for the estimation of heritability of a trait (Fehr, 1987). Broad-sense heritability was recorded very high for traits such as plant height, number of leaves, leaf diameter, pseudostem length, pseudostem width, neck thickness, total soluble solids, dry matter content, bolting percentage, average bulb weight, days to maturity, and PDI, and results are in agreement with others (Khosa and Dhatt, 2013; Santra et al., 2017; Dangi et al., 2018; Bagchi et al., 2020).

Out of 157 genotypes belonging to the *Allium cepa* group, 16 were classified as moderately resistant. Among them, genotype SBO113 exhibited the lowest percent disease incidence. None of the genotypes belonging to the *Allium cepa* group was found to be immune or completely resistant against *Stemphylium* leaf blight. Most of the genotypes were found to be moderately susceptible or susceptible, and only one genotype was classified as highly susceptible, i.e., SBO131. In previous studies also, only moderate resistance was reported in *Allium cepa* and none of the accessions were found to be completely resistant against *Stemphylium* leaf blight (Pathak et al., 2001; Behera et al., 2013). Dangi et al. (2019) screened 57 onion accessions against *Stemphylium* leaf blight and reported one onion accession ‘Red Creole 2’ as moderately resistant and rest were susceptible.

Pearson’s correlation coefficient depicted a significantly positive correlation between total soluble solids and dry matter content, which is in conformity with the observation of other authors (Jaime et al., 2001; Dangi et al., 2019). A significant correlation between soluble solids and dry matter content has been reported by other researchers also (Nieuwhof et al., 1973; Sinclair et al., 1995). A highly significant negative correlation was observed for dry matter content and PDI. Dangi et al. (2019) also demonstrated a significant negative correlation between PDI and dry matter content. A negative correlation of traits such as leaf length, number of leaves, and pseudostem width with PDI was also observed. Positive and significant correlations between equatorial diameter, polar diameter, and average bulb weight have been observed by other researchers in onion (Aliyu et al., 2007; Singh et al., 2010). Results of correlation coefficients are in agreement with Trivedi and Dhumal (2010); Dewangan and Sahu (2014) for the correlations between average bulb weight and marketable yield.

In the current study, genotypes having higher total foliar phenols showed slower disease progression which ultimately resulted in lower infection as compared with genotypes which have lower total phenolic contents upon inoculation under controlled conditions. The accumulation of phenolic compounds at the infection site may obstruct the disease infection or slow down pathogen spread due to the toxic activity of these compounds against pathogens or by increasing the mechanical strength of the host cell wall (Benhamou et al., 2000; Dangi et al., 2019). Also, the higher phenolic acid levels may cause changes in pH of plant cell cytoplasm, which results in the inhibition of pathogen development and ultimately enhance the level of resistance (Ojalvo et al., 1987; Mahmoodi-Khaledi et al., 2015). Medina-Melchor et al. (2022) reported that upon infection with *S. vesicarium*, the concentration of pyruvic acid increased in the bulb, roots, and leaves of onion plants. Likewise, in our study also pyruvic acid in leaf tissue increased from before inoculation and was found maximum at 20 days after inoculation in genotypes having low disease incidence. Pyruvic acid can be utilized by *S. vesicarium* as a source of nutrition for its growth. The genotypes with lower incidence were able to resist the *Stemphylium vesicarium* proliferation due to higher pyruvic acid concentrations. In genotypes with high incidence, the fungus proliferated profusely by utilizing pyruvic acid, which acts as source of nitrogen, ultimately resulting in lower pyruvic acid content. Infection with *Magnaporthe grisea* also leads to an increase in pyruvic acid in host plant species after 3 days. Parker et al. (2009) suggested that this increase in pyruvic acid and other alterations in the carbohydrate metabolism and TCA cycle allow the growth of pathogens in living plant cells. In fungi, the destination of pyruvate is the TCA cycle in mitochondria, which is the energy-yielding metabolic route for ATP production by oxidative phosphorylation (Walker and White, 2017).

In the context of enzyme evaluation, the catalase activity exhibited an increase in its accumulation before inoculation to 20 days after inoculation. An increment of 2.5-fold has been recorded before inoculation to 20 days after inoculation in genotypes which have lower PDI values, and thereafter it declined. The increment of catalase was sharp in genotypes with lower PDI values as compared with genotypes with higher PDI values. This increase in the activity of the catalase enzyme in genotypes (SBO113) having the lowest PDI values might be due to the natural defense response of the genotype. The infection caused by plant pathogens leads to excess H_2_O_2_ production in the cell peroxisomes because the oxidases are involved in the oxidation of fatty acids, photorespiration, and purine catabolism (Ahmad et al., 2010). Toxicity due to higher levels of H_2_O_2_ can result in plant cell damage, whereas at lower concentrations, it plays a very important role in signal transduction in the plant which is under attack (Scandalios et al., 2000; Pittner et al., 2019). However, under the action of catalase, it is converted to H_2_O and O_2_ and prevents the further cell damage (Debona et al., 2012). Several studies reported the role of the peroxidase enzyme in plant resistance mechanism against pathogens by eliminating the reactive oxygen species and catalyzing the oxidoreduction of various substrates using hydrogen peroxide (Kawaoka et al., 2003; Almagro et al., 2009). The results pertaining to peroxidase enzyme in the present study recorded a steady increase irrespective of the genotypes, up to 20 days. Enzyme activity was recorded almost 1.5 times higher among genotypes having lower values for disease incidence, as compared with genotypes having higher disease incidence values. Plant pathogen infection stimulated the peroxidase accumulation in plants upon infection, which was recorded higher in resistant plants compared with the susceptible ones (Mydlarz and Harvell, 2007). These reports are in agreement with the present study findings, which showed that the peroxidase activity was maintained at a higher level in the leaves of genotypes with lower disease incidence. The involvement of total protein content in plant defense mechanisms against fungi and bacteria has also been reported among different crops (Carvalho et al., 2006; Ashfaq et al., 2021). In the current study, a decreasing trend in total protein content irrespective of the genotypes as disease progresses was observed. However, the rate of total protein content decline was higher in genotypes with higher disease incidence. Proteins being a major component of the plant cell cannot explain the total variation in phenotype, but it may be associated in the host defense mechanism against pathogens (Ashfaq et al., 2021). In previous reports also, phenolic compounds, pyruvic acid, and antioxidant enzymes such as catalase and guaiacol peroxidase were found higher in *Alternaria porri*-infected plants as compared with control (Khandagale et al., 2022). In the current study, total foliar phenolic content was found to be positively correlated with pyruvic acid content, protein content, and antioxidant enzymes (CAT and POX). Higher phenolic content may induce the production of host pathogenesis-related (PR) proteins, which prevent the reproduction and further spread of pathogen in infected leaf tissue (Ozdal et al., 2013; Awan et al., 2018). A significant positive correlation was also recorded in between catalase, peroxidase, and protein under disease stress (Awan et al., 2018).

It was also observed that the PDI of moderately resistant genotypes under stage I was categorized as susceptible in stage II. This depicts that the screening of onions should be done separately at both the seed-to-bulb stage (stage I) and bulb-to-seed stage (stage II). Accessions which are categorized as resistant in stage I may or may not be resistant in stage II. SLB attacks onions in both the stages. Hence, a screening strategy to identify resistant accessions in both the stages is important.

## Conclusion

Onions are the second most important vegetable crop after tomato used throughout the world for food and medicinal purposes. Production and productivity losses due to biotic and abiotic factors impede the economic survivability of farmers. *Stemphylium* leaf blight has emerged as a major foliar disease affecting 90% of the bulb and seed crops in the epidemic state. Management of SLB through fungicides is a short-term strategy. Moreover, it leads to resistance buildup in pathogens and loss of foraging insects. The sustainable way is to develop resistant varieties that are in harmony with nature. Screening of 157 onion genotypes resulted in the identification of 15 moderately resistant genotypes in stage I. In stage II, these selected genotypes were screened again to confirm their resistant status. Although the selected genotypes were able to withstand the disease, they were categorized as susceptible. Genotypes characterized as susceptible or highly susceptible in stage I recorded an almost similar reaction in stage II also. Our hypothesis is that the identification of SLB-resistant onions should be done at two stages: one at the seed-to-bulb formation stage (stage I) and other screening at bulb-to-seed stage (stage II). Screening at one stage will not be sufficient to identify the *Stemphylium* leaf blight-resistant material. In morphological traits, leaf length, number of leaves, pseudostem width, and neck thickness showed a significantly negative correlation and selection of genotypes with lower values for these traits should be selected. High dry matter, phenol content, pyruvic acid, peroxidase, catalase, and protein content are the potential biochemical traits for identification of tolerant/resistant genotypes. These traits can serve as biomarkers for high-throughput screening of genotypes at the initial phase of the resistance breeding program. A comprehensive strategy involving the identified morphological and biochemical traits in stage I and stage II will be an effective strategy to devise an SLB-resistant program in onions.

## Data availability statement

The original contributions presented in the study are included in the article/**Supplementary Material**. Further inquiries can be directed to the corresponding author.

## Author contributions

RC and AKh conceived the idea, designed the methodology, and wrote the manuscript. RC, DK, AKh, and SS designed the methodology and helped in screening experiment. RC, MZ, and PV participated in field works and indoor experiments. AKu, RP, and AKh analyzed the data and results. RC, RP, and AKh produced the figures and tables. All authors contributed equally to the article and approved the submitted version.

## Acknowledgments

The authors are highly thankful to the Head, Division of Vegetable Science, ICAR-Indian Agricultural Research Institute, New Delhi, for providing all sorts of facilities and financial support for this study. The first author also wants to acknowledge the ICAR-Indian Agricultural Research Institute for awarding Senior Scholarship during this period of research work.

## Conflict of interest

The authors declare that the research was conducted in the absence of any commercial or financial relationships that could be construed as a potential conflict of interest.

## Publisher’s note

All claims expressed in this article are solely those of the authors and do not necessarily represent those of their affiliated organizations, or those of the publisher, the editors and the reviewers. Any product that may be evaluated in this article, or claim that may be made by its manufacturer, is not guaranteed or endorsed by the publisher.

## References

[B1] Aebi H. (1984). Catalase *in vitro* . Methods Enzymol. 105, 121–126. doi: 10.1016/S0076-6879(84)05016-3 6727660

[B2] Ahmad P. Jaleel C. A. Salem M. A. Nabi G. Sharma S. (2010). Roles of enzymatic and nonenzymatic antioxidants in plants during abiotic stress. Crit. Rev. Biotechnol. 30, 161–175. doi: 10.3109/07388550903524243 20214435

[B3] Aliyu U. Magaji M. D. Singh A. Mohammed S. G. (2007). Growth and yield of onion (*Allium cepa* l.) as influenced by nitrogen and phosphorus levels. Int. J. Agric. Sci. 2, 937–944. doi: 10.3923/ijar.2007.937.944

[B4] Almagro L. Gómez Ros L. V. Belchi-Navarro S. Bru R. Ros Barceló A. Pedreño M. A. (2009). Class III peroxidases in plant defense reactions. J. Exp. Bot. 60, 377–390. doi: 10.1093/jxb/ern277 19073963

[B5] Anthon G. E. Barrett D. M. (2003). Modified method for the determination of pyruvic acid with dinitrophenylhydrazine in the assessment of onion pungency. J. Sci. Food Agric. 83, 1210–1213. doi: 10.1002/jsfa.1525

[B6] APEDA (2020) India Export of agro-food products. Available at: http://agriexchange.apeda.gov.in/indexp/Product_description_32head.aspx?gcode=0202 (Accessed October 06, 2022).

[B7] Arya J. S. Singh N. Arya P. Kant A. (2017). Morphological variations and relationship among onion germplasm for quantitative and qualitative traits at trans-himalaya ladakh, India. Aust. J. Crop Sci. 11, 329–337. doi: 10.21475/ajcs.17.11.03.pne369

[B8] Ashfaq B. Arshad H. M. I. Atiq M. Yousaf S. Saleem K. Arshad A. (2021). Biochemical profiling of resistant phenotypes against bipolaris oryzae causing brown spot disease in rice. Front. Agron 3, 675895. doi: 10.3389/fagro.2021.675895

[B9] Awale D. Sentayehu A. Getachew T. (2011). Genetic variability and association of bulb yield and related traits in shallot (Allium cepa var. aggregatum don.) in Ethiopia. Int. J. Agric. Res. 6, 517–536. doi: 10.3923/ijar.2011.517.536

[B10] Awan Z. A. Shoaib A. Khan K. A. (2018). Variations in total phenolics and antioxidant enzymes cause phenotypic variability and differential resistant response in tomato genotypes against early blight disease. Sci. Hortic. 239, 216–223. doi: 10.1016/J.SCIENTA.2018.05.044

[B11] Bagchi C. K. Shree S. Ansar M. Saxena A. S. Kumari M. (2020). Polygenic variations and character association of morphological, biochemical and disease related traits in garlic (*Allium sativum* l.). J. pharmacogn. phytoch. 9, 1277–1283.

[B12] Basallote-Ureba M. J. Prados-Ligero A. M. Melero-Vara J. M. (1999). Aetiology of leaf spot of garlic and onion caused by stemphylium vesicarium in Spain. Plant Pathol. 48, 139–145. doi: 10.1046/j.1365-3059.1999.00313.x

[B13] Behera S. Santra P. Chattopadhyay S. Das S. Maity T. K. (2013). Variation in onion varieties for reaction to natural infection of *Alternaria porri* (Ellis) ciff. and *Stemphylium vesicarium* (Wallr.). Bioscan 8, 759–761.

[B14] Benhamou N. Gagné S. Le Quéré D. Dehbi L. (2000). Bacterial-mediated induced resistance in cucumber: beneficial effect of the endophytic bacterium *Serratia plymuthica* on the protection against infection by *Pythium ultimum* . Phytopathology 90, 45–56. doi: 10.1094/PHYTO.2000.90.1.45 18944571

[B15] Bradford M. M. (1976). A rapid and sensitive method for the quantitation of microgram quantities of protein utilizing the principle of protein-dye binding. Anal. Biochem. 72, 248–254. doi: 10.1006/abio.1976.9999 942051

[B16] Brown J. K. M. (2006). “Surveys of variation in virulence and fungicide resistance and their application to disease control,” in The epidemiology of plant diseases. Eds. Cooke B. M. Jones D. G. Kaye B. (Dordrecht: Springer), 81–115.

[B17] Burton G. W. Devane D. E. (1953). Estimating heritability in tall fescue (*Festuca arundinacea*) from replicated clonal material 1. Agron. J. 45, 478–481. doi: 10.2134/agronj1953.00021962004500100005x

[B18] Carvalho D. D. Ferreira R. A. Oliveira L. M. D. Oliveira A. F. D. Gemaque R. C. R. (2006). Proteins and isozymes electroforesis in seeds of *Copaifera langsdorffii* desf. (*leguminosae caesalpinioideae*) artificially aged. Rev. Árvore. 30, 19–24. doi: 10.1590/S0100-67622006000100003

[B19] Chance B. Maehly A. E. (1955). “Assay of catalase and peroxidase,” in Methods in enzymology. Eds. Colowick S. P. Kaplan N. O. (New York: Academic Press, Inc), 764–775.

[B20] Chattopadhyay A. Sharangi A. B. Dutta S. Das S. Denre M. (2013). Genetic relatedness between quantitative and qualitative parameters in onion (*Allium cepa* l.). Vegetos-An Int. J. Plant Res. 26, 151–157. doi: 10.5958/j.2229-4473.26.1.021

[B21] Cullings K. W. (1992). Design and testing of a plant-specific PCR primer for ecological and evolutionary studies. Mol. Ecol. 1, 233–240. doi: 10.1111/j.1365-294X.1992.tb00182.x

[B22] Dangi R. Kumar A. Khar A. (2018). Genetic variability, heritability, and diversity analysis studies in short day tropical onion (*Allium cepa* l.). Indian. J. Agric. Sci. 88, 948–957. doi: 10.1007/s13313-019-00639-x

[B23] Dangi R. Sinha P. Islam S. Gupta A. Kumar A. Rajput L. S.. (2019). Screening of onion accessions for stemphylium blight resistance under artificially inoculated field experiments. Australas. Plant Pathol. 48, 375–384. doi: 10.1007/s13313-019-00639-x

[B24] Debona D. Rodrigues F. A. Rios J. A. Nascimento K. J. T. (2012). Biochemical changes in the leaves of wheat plants infected by *Pyricularia oryzae* . Phytopathology 102, 1121–1129. doi: 10.1094/PHYTO-06-12-0125-R 22913412

[B25] Deshmukh S. N. Basu M. S. Reddy P. S. (1986). Genetic variability, character association and path coefficients of quantitative traits in Virginia bunch varieties of groundnut. Indian. J. Agric. Sci. 56, 816–821.

[B26] Dewangan S. R. Sahu G. D. (2014). Genetic variability, correlation and path coefficient analysis of different kharif onion genotypes in chhattisgarh plains. Agric. Sci. Digest. 34, 233–236. doi: 10.5958/0976-0547.2014.01010.6

[B27] Ellis M. B. (1971). Dematiaceous hyphomycetes (Kew, Surrey, UK: Commonwealth Mycological Institute), 608.

[B28] FAOSTAT (2020) Onion production, area and productivity. Available at: http://faostat3.fao.org/browse/Q/QC/E (Accessed October 06, 2022).

[B29] Fehr R. W. (1987). “Heritability,” in Principles of cultivar development (New York: Macmillian Publishing Company), 95–96.

[B30] Foster J. M. Tayviah C. S. Stricker S. M. Gossen B. D. McDonald M. R. (2019). Susceptibility to *Stemphylium vesicarium* of asparagus, onion, pear, and rye in Canada. Can. J. Plant Pathol. 41, 228–241. doi: 10.1080/07060661.2019.1574901

[B31] Gedefaw Y. Gezahegn A. Fekadu A. Mehari Z. (2019). First report of *Stemphylium vesicarium* causing onion stemphylium leaf blight in Ethiopia. Agric. Sci. 10, 1104. doi: 10.4236/as.2019.108083

[B32] Gillespie S. Long R. Seitz N. Williams N. (2014). Insecticide use in hybrid onion seed production affects pre-and post pollination processes. J. Econ. Entomol. 107, 29–37. doi: 10.1603/ec13044 24665681

[B33] Hassan M. Yousuf V. Bhat Z. A. Bhat N. A. Shah T. A. Khan M. A.. (2020). Morpho-cultural and pathogenic variability among isolates of *Stemphylium vesicarium* (Wallr.) e. Simmons, causing stemphylium blight in onion collected from different geographical regions of Kashmir valley. Indian Phytopathol. 73, 469–481. doi: 10.1007/s42360-020-00253-8

[B34] Hay F. S. Sharma S. Hoepting C. Strickland D. Luong K. Pethybridge S. J. (2019). Emergence of stemphylium leaf blight of onion in new York associated with fungicide resistance. Plant Dis. 103, 3083–3092. doi: 10.1094/PDIS-03-19-0676-RE 31596693

[B35] Hay F. Stricker S. Gossen B. D. McDonald M. R. Heck D. Hoepting C.. (2021). Stemphylium leaf blight: A re-emerging threat to onion production in eastern north America. Plant Dis. 105, 3780–3794. doi: 10.1094/PDIS-05-21-0903-FE 34546780

[B36] Hoepting C. A. (2017). Effect of fungicide timing on stemphylium leaf blight on onion 2016. Plant Dis. Manage. Rep. 11, V130.

[B37] Hussein M. Abo-Elyousr K. A. Hassan M. A. Hashem M. Hassan E. A. Alamri S. A. (2018). Induction of defense mechanisms involved in disease resistance of onion blight disease caused by *Botrytis allii* . Egypt J. Biol. Pest Control 28, 1–11. doi: 10.1186/s41938-018-0085-5

[B38] Jaime L. Martínez F. Martín-Cabrejas M. A. Mollá E. López-Andréu F. J. Waldron K. W.. (2001). Study of total fructan and fructooligosaccharide content in different onion tissues. J. Sci. Food Agric. 81, 177–182. doi: 10.1002/1097-0010(20010115)81:2<177::AID-JSFA796>3.0.CO;2-9

[B39] Javadzadeh A. Ghorbanihaghjo A. Bonyadi S. Rashidi M. R. Mesgari M. Rashtchizadeh N.. (2009). Preventive effect of onion juice on selenite-induced experimental cataract. Indian J. Ophthalmol. 57, 185. doi: 10.4103/0301-4738.49391 19384011 PMC2683439

[B40] Kawaoka A. Matsunaga E. Endo S. Kondo S. Yoshida K. Shinmyo A.. (2003). Ectopic expression of a horseradish peroxidase enhances growth rate and increases oxidative stress resistance in hybrid aspen. Plant Physiol. 132, 1177–1185. doi: 10.1104/pp.102.019794 12857800 PMC167058

[B41] Khandagale K. Roylawar P. Kulkarni O. Khambalkar P. Ade A. Kulkarni A.. (2022). Comparative transcriptome analysis of onion in response to infection by *Alternaria porri* (Ellis) cifferi. Front. Plant Sci. 13, 857306. doi: 10.3389/fpls.2022.857306 35481153 PMC9036366

[B42] Khar A. Galván G. A. Singh H. (2022). “Allium breeding against biotic stresses,” in Genomic designing for biotic stress resistant vegetable crops. Ed. Kole C. (Cham: Springer). doi: 10.1007/978-3-030-97785-6_6

[B43] Khosa J. S. Dhatt A. S. (2013). Studies on genetic variability and heritability in bulb onion (*Allium cepa* l.) in north-Western plains of India. J. Hortic. Sci. 8, 255–258.

[B44] Koul O. Walia S. W. (2009). Comparing impacts of plant extracts and pure allelochemicals and implications for pest control. CAB Rev. 4, 1–30. doi: 10.1079/PAVSNNR20094049

[B45] Kumar P. (2007). Genetics of resistance to stemphylium leaf blight of lentil (Lens culinaris) in the barimasur-4 × CSC milestone (Sakatchewan, (CA: University of Saskatchewan).

[B46] Lorbeer J. W. (1993). “A serious outbreak of stemphylium leaf blight of onion in new York,” in Proceedings of the 1993 national onion research conference (Ithaca: Cornell University), 32–37.

[B47] Madden L. V. Hughes G. Van Den Bosch F. (2007). “The study of plant disease epidemics,” in The American phytopathological society (St. Paul. Minnesota: APS Press).

[B48] Mahmoodi-Khaledi E. Kashef N. Habibi-Rezaei M. Moosavi-Movahedi A. A. (2015). *In vitro* characterization of antibacterial potential of Iranian honey samples against wound bacteria. Eur. Food Res. Technol. 241, 329–339. doi: 10.1007/s00217-015-2464-4

[B49] Mallor Giménez C. Carravedo Fantova M. Estopañán Muñoz G. Mallor Giménez F. (2011). Characterization of genetic resources of onion (*Allium cepa* l.) from the Spanish secondary centre of diversity. Span. J. Agric. Res. 9, 144–155. doi: 10.5424/sjar/20110901-149-10

[B50] McArt S. H. Urbanowicz C. McCoshum S. Irwin R. E. Adler L. S. (2017). Landscape predictors of pathogen prevalence and range contractions in US bumblebees. Proc. R. Soc Biol. Sci. 284, 20172181. doi: 10.1098/rspb.2017.2181 PMC5719184 29142119

[B51] Medina-Melchor D. L. Zapata-Sarmiento D. H. Becerra-Martínez E. Rodríguez-Monroy M. Vallejo L. Sepúlveda-Jiménez G. (2022). Changes in the metabolomic profiling of *Allium cepa* l. (onion) plants infected with *Stemphylium vesicarium* . Eur. J. Plant Pathol. 162, 557–573. doi: 10.1007/s10658-021-02421-6

[B52] Miller M. E. Taber R. A. Amador J. M. (1978). Stemphylium blight of onion in south Texas. Plant Dis. 62, 851–853.

[B53] Mydlarz L. D. Harvell C. D. (2007). Peroxidase activity and inducibility in the sea fan coral exposed to a fungal pathogen. *Comp. biochem. physiol.* part a. Mol. Integr. Physiol. 146, 54–62. doi: 10.1016/j.cbpa.2006.09.005 17064941

[B54] Nieuwhof M. De Bruyn J. W. Garretsen F. (1973). Methods to determine solidity and dry matter content of onions (*Allium cepa* l.). Euphytica 22, 39–47. doi: 10.1007/BF00021554

[B55] Ojalvo I. Rokem J. S. Navon G. Goldberg I. (1987). 31P NMR study of elicitor treated *Phaseolus vulgaris* cell suspension cultures. Plant Physiol. 85, 716–719. doi: 10.1104/pp.85.3.716 16665766 PMC1054328

[B56] Ozdal T. Capanoglu E. Altay F. (2013). A review on protein–phenolic interactions and associated changes. Food Res. Int. 51, 954–970. doi: 10.1016/j.foodres.2013.02.009

[B57] Paibomesai M. Celetti M. Tesfaendrias M. (2012). Update on stemphylium leaf blight of onions in Ontario. Hort Matters 12, 11–12.

[B58] Parker D. Beckmann M. Zubair H. Enot D. P. Caracuel-Rios Z. Overy D. P.. (2009). Metabolomic analysis reveals a common pattern of metabolic re-programming during invasion of three host plant species by *Magnaporthe grisea* . Plant J. 59, 723–737. doi: 10.1111/j.1365-313X.2009.03912.x 19453445

[B59] Pathak C. S. Black L. L. Cherng S. J. Wang T. C. Ko S. S. (2001). Breeding onions for stemphylium leaf blight resistance. Acta Hortic. 555, 77–81. doi: 10.17660/ActaHortic.2001.555.7

[B60] Pathak C. S. Singh A. Despande A. Sridar T. T. (1986). Source of resistance to purple blotch in onion. Veg. Sci. 13, 300–303.

[B61] Pittner E. Marek J. Bortuli D. Santos L. A. Knob A. Faria C. M. D. R. (2019). Defense responses of wheat plants (*Triticum aestivum* l.) against brown spot as a result of possible elicitor’s application. Arq. Inst. Biol. (Sao Paulo) 86, 1–16. doi: 10.1590/1808-1657000312017

[B62] Popat R. Patel R. Parmar D. (2020) Variability: GeneticVariability analysis for plant breeding research. Available at: https://cran.r-project.org/web/packages/variability/variability.pdf.

[B63] Raghavendra Rao N. N. Pavgi M. S. (1975). Stemphylium leaf blight of onion. Mycopathologia 56, 113–118. doi: 10.1007/BF00472582

[B64] RStudio (2021). RStudio: Integrated development environment for r (Boston, MA: RStudio, PBC). Available at: http://www.rstudio.com/.

[B65] Santra P. Manna D. Sarkar H. K. Maity T. K. (2017). Genetic variability, heritability and genetic advance in *kharif* onion (*Allium cepa* l.). J. Crop Weed 13, 103–106.

[B66] Scandalios J. G. Acevedo A. Ruzsa S. (2000). Catalase gene expression in response to chronic high temperature stress in maize. Plant Sci. 156, 103–110. doi: 10.1016/S0168-9452(00)00235-1 10908810

[B67] Sharma S. R. (1986). Effect of fungicidal sprays on purple blotch and bulb yield of onion. Indian Phytopathol. 39, 78–82.

[B68] Sharma S. N. Sain R. S. (2003). RO-1 an improved onion variety for the warmer areas of rajasthan. Indian J. Genet. Pl. Br. 63, 281–282.

[B69] Sinclair P. J. Blakeney A. B. Barlow E. W. R. (1995). Relationships between bulb dry matter content, soluble solids concentration and non-structural carbohydrate composition in the onion (*Allium cepa*). J. Sci. Food Agric. 69, 203–209. doi: 10.1002/jsfa.2740690210

[B70] Singh B. (2001). Plant breeding: Principles and methods. 6th ed (New Delhi, India: Kalyani Publishers).

[B71] Singh D. Dhiman J. S. Sidhu A. S. Singh H. (1992). Current status of onions in India: strategies for disease resistance breeding for sustained production. Onion News. Topics. 4, 43–44.

[B72] Singh R. K. Dubey B. K. Bhonde S. R. Gupta R. P. (2010). Estimates of genetic variability and correlation in red onion (*Allium cepa*) advance lines. Indian J. Agric. Sci. 80, 160–163.

[B73] Singleton V. L. Rossi J. A. (1965). Colorimetry of total phenolics with phosphomolybdic-phosphotungstic acid reagents. Am. J. Enol. Vitic. 16, 144–158.

[B74] Solanki P. Jain P. K. Prajapati S. Raghuwanshi N. Khandait R. N. Patel S. (2015). Genetic analysis and character association in different genotypes of onion (*Allium cepa* l.). Int. J. Agric. Environ. Biotechnol. 8, 783. doi: 10.5958/2230-732X.2015.00087.X

[B75] Suheri H. Price T. V. (2000). Infection of onion leaves by *Alternaria porri* and *Stemphylium vesicarium* and disease development in controlled environments. Plant Pathol. 49, 375–382. doi: 10.1046/j.1365-3059.2000.00458.x

[B76] Tomaz I. Lima A. (1988). An important disease of onion caused by *Stemphylium vesicarium* (Wallr.) Simmons in Portugal. Hortic. Abstr. 68, 618.

[B77] Trivedi A. P. Dhumal K. N. (2010). Variability and correlation studies on bulb yield, morphological and storage characters in onion (*Allium cepa* l.). J. Pure Appl. Sci. 18, 1–4.

[B78] Walker G. M. White N. A. (2017). “Introduction to fungal physiology,” in Fungi: biology and applications. Ed. Kavanagh K. (USA: John Wiley & Sons, Inc), 1–35.

[B79] Wheeler B. E. J. (1969). An introduction to plant diseases (New York: John Wiley & Sons Ltd).

[B80] White T. J. Bruns T. Lee S. J. Taylor J. L. (1990). “Amplification and direct sequencing of fungal ribosomal RNA genes for phylogenetics,” in PCR protocols: a guide to methods and applications Eds. Innis M. A. Gelfand D. H. Sninsky J. J. White T. J. (New York: Academic Press Inc,), 315–322.

[B81] Zhao X. X. Lin F. J. Li H. Li H. B. Wu D. T. Geng F.. (2021). Recent advances in bioactive compounds, health functions, and safety concerns of onion (*Allium cepa* l.). Front. Nutr. 8. doi: 10.3389/fnut.2021.669805 PMC8339303 34368207

