# Supplementary material for: Screening of short-day onions for resistance to Stemphylium leaf blight in the seed-to-bulb stage (stage I) and bulb-to-seed stage (stage II)
Source: Front Plant Sci. 2022 Nov 16;13:1063685. doi: 10.3389/fpls.2022.1063685 (PMC9709266; doi:10.3389/fpls.2022.1063685)
Supplement: Supplementary file 1 [file Table_1.docx]

Supplementary Table 1 : List of onion genotypes used in the study with parentage, status, origin and breeding institute

Supplementary Table 2: Morphological, biochemical traits, PDI, AUDPC and disease reaction of 157 onion genotypes during Stage I

Supplementary table 3 : PDI (Percent Disease Incidence) during 10, 20 and 30 days after inoculation (DAI) and cumulative AUDPC (Area Under Disease Progress Curve)
